# Substrate pH Influences the Nutrient Absorption and Rhizosphere Microbiome of Huanglongbing-Affected Grapefruit Plants

**DOI:** 10.3389/fpls.2022.856937

**Published:** 2022-05-13

**Authors:** Rhuanito Soranz Ferrarezi, Xiongjie Lin, Andres C. Gonzalez Neira, Flavia Tabay Zambon, Hanqing Hu, Xianda Wang, Jing-Hao Huang, Guocheng Fan

**Affiliations:** ^1^Department of Horticulture, University of Georgia, Athens, GA, United States; ^2^Horticultural Sciences Department, Indian River Research and Education Center, Institute of Food and Agricultural Sciences, University of Florida, Fort Pierce, FL, United States; ^3^Fruit Research Institute, Fujian Academy of Agricultural Sciences/Fujian Key Laboratory of Plant Nutrition and Fertilizer, Fuzhou, China; ^4^Institute of Plant Protection, Fujian Academy of Agricultural Sciences, Fuzhou, China

**Keywords:** mineral nutrition, photosynthesis, root-to-shoot ratio, 16S rRNA, root microbiome, controlled environment

## Abstract

The substrate pH directly affects nutrient availability in the rhizosphere and nutrient uptake by plants. Macronutrients such as nitrogen, potassium, calcium, magnesium, and sulfur are highly available at pH 6.0–6.5, while micronutrients become less available at higher, alkaline pH (pH > 7.0). Recent research has indicated that low pHs can enhance nutrient uptake and improve sweet orange (*Citrus sinensis*) tree health. We designed a study to understand the influence of a wide range of substrate pH values on plant size and biomass, nutrient availability, leaf gas exchange, and rhizosphere microbiome of grapefruit (*Citrus paradisi*) affected by Huanglongbing (HLB). Two-year-old “Ray Ruby” grapefruit plants grafted on sour orange (*Citrus aurantium*) rootstock were cultivated indoors in 10-cm wide × 40-cm tall pots with peat:perlite commercial substrate (80:20 v/v). We tested two disease statuses [HLB-free or healthy (negative, HLB–) and HLB-affected (positive, HLB+)] and six substrate pH values (4, 5, 6, 7, 8, 9) in a 2 × 6 factorial arranged on a complete randomized design with four replications. The canopy volume of HLB+ plants was 20% lower than healthy plants, with pHs 7 and 9 resulting in 44% less canopy volume. The root and shoot ratio of dry weight was 25.8% lower in HLB+ than in healthy plants. Poor root growth and a decrease in fibrous roots were found, especially in pH 5 and 6 treatments in HLB+ plants (*p* < 0.0001). The disease status and the substrate pHs influenced the leaf nutrient concentration (*p* < 0.05). High substrate pH affects nutrient availability for root uptake, influencing the nutrient balance throughout the plant system. pH values did not affect plant photosynthesis, indicating that pH does not recover HLB+ plants to the photosynthetic levels of healthy plants—even though high pH positively influenced internal CO_2_. There were collectively over 200 rhizobacterial identified by the 16S rRNA gene sequencing in individual phylogenetic trees. Most rhizobacteria reads were identified in pH 9. Our results indicated no effect of substrate pHs on the plant disease status induced by enhanced nutrient uptake.

## Introduction

Citrus greening or Huanglongbing (HLB) is one of the most destructive diseases of citrus, associated with the phloem-limited bacterium *Candidatus* Liberibacter asiaticus (*C*Las) and transmitted by the Asian citrus psyllid (ACP, *Diaphorina citri*).

The disease impacts all major citrus production countries differently due to their marketing and production strategies. The United States has faced a dwindling citrus production and acreage over the last 17 years since all citrus varieties are susceptible to the bacteria. In particular, the grapefruit (*Citrus paradisi*) production for the fresh market is remarkedly affected by this devastating disease, reduced by 80% from 2.5 million tons in 2000–2001 to 500,000 tons in 2019–2020. Grapefruit growers were drastically impacted by the sharp decline in production, reducing the commercial acreage from 107,800 acres to 21,700 acres in 2019–2020 (U. S Department of Agriculture, 2021).

Although HLB was first reported more than 100 years ago, resistant varieties and successful control methods are unavailable (Bové, 2006). To manage the disease and stay in business, growers from around the world rely on multiple strategies to cope with the disease, by using HLB-free nursery plants, chemical spraying to control the insect vector ACP, eradication of infected trees in the field, and application of effective management practices (Xia et al., 2011).

Mineral nutrients are vital for plant development and are indispensable factors in plant–disease interactions, as their presence could create a less favorable environment for disease development (Spann and Schumann, 2009). Trace elements are required in small amounts for plant growth and are critical cofactors in metabolic processes. Boron (B) and zinc (Zn) are examples of essential elements of higher plants, participating in several biochemical and physiological processes such as nitrogen (N) fixation, cellular respiration, and photosynthesis. Symptomatic HLB+ leaves are known for having lower iron (Fe) and Zn concentrations than healthy leaves (Masaoka et al., 2011), mimicking the blotchy mottled pattern of the classic visual HLB symptom. An alternative to supply HLB+ trees with enough nutrients is to apply targeted foliar sprays with distinct concentrations of macro and micronutrients. Shen et al. (2013) showed that foliar applications of a mixture of fertilizers, biological pesticides, and systemic resistance inducing agents increased leaf calcium (Ca), B, manganese (Mn), and Zn concentrations and reduced *C*Las titer in HLB+ trees. Leaf phosphorus (P), potassium (K), magnesium (Mg), and Fe concentrations remained unchanged, while leaf copper (Cu) concentrations decreased. The leaf N, Mg, and Fe concentrations were 12, 21, and 42% lower in HLB+ trees than healthy trees cultivated in sandy and clay–loam soil types subjected to different fertilizer treatments (Pustika et al., 2008). Such discrepancy is caused by a poor root development of HLB+ trees, restricting nutrient uptake and transport of minerals, influencing leaf mineral concentrations. Foliar fertilizer application reduces disease symptoms as the plant utilizes foliar-applied minerals locally in several defense pathways, prolonging tree life and reducing yield losses (Pustika et al., 2008).

Alkaline soil and water conditions increase the loss of feeder roots in HLB+ sweet orange trees and affect their performance regardless of disease occurrence (Morgan and Graham, 2019; Ghimire et al., 2020). Thus, maintaining the topsoil root zone pH in the 5.5–6.5 range is crucial for root development, as fertilizer management decreases soil pH over time and soil pH increases with the soil depth (Atta et al., 2020). Acidic soils can increase the root metabolic activity and up-regulate the expression of ion transporter genes in HLB+ roots, the expression of genes involved in systemic acquired resistance, and the salicylic acid signaling pathway, alleviating physiological disorders caused by the phloem sieve tube blockage (Li et al., 2020).

The rhizosphere is responsible for water and nutrient uptake and provides a viable niche for microbial activity proliferation in exchange for various nutritional and metabolic processes. The rhizosphere is replete with plant-derived compounds that would be likely used as nutrient sources or elicitors for the microbes that enhance defense responses in plants against pests and diseases. The predominant taxa of the microbiome in the rhizosphere zone include Proteobacteria, Actinobacteria, Acidobacteria, and Bacteroidetes. In HLB+ trees, the unbalanced phloem load/unloading process caused by blockage of the sieve tubes inhibits nutrient uptake by physiologically modifying the root endosphere that interacts with the rhizosphere (Zhang et al., 2017). Research focusing on the interaction between HLB and the growing media pH in grapefruit plant growth and microbiome is still scarce. Nutrient availability studies are crucial to establishing the pathways for plant and microbe interactions in the rhizosphere.

This study subjected healthy and HLB+ grapefruit plants to a wide range of substrate pH values and evaluated their effects on plant growth performance and rhizosphere microorganism population diversity. Our hypothesis is that acidic substrate conditions will increase micronutrient availability and plant photosynthesis, accelerate plant growth, and create a more diverse and dynamic rhizosphere microorganism microbiome, allowing the plant to cope with the negative effects of HLB.

## Materials and Methods

### Study Location and Environmental Conditions

This study was conducted from 27 August, 2019 (Week 0) to 14 January, 2020 (Week 20) at the University of Florida/Institute of Food and Agricultural Sciences (UF/IFAS) Indian River Research and Education Center in Fort Pierce, Florida, United States (27°25'35'N, 80°24'31” W, 5.8 m elevation).

**Figure 1:**
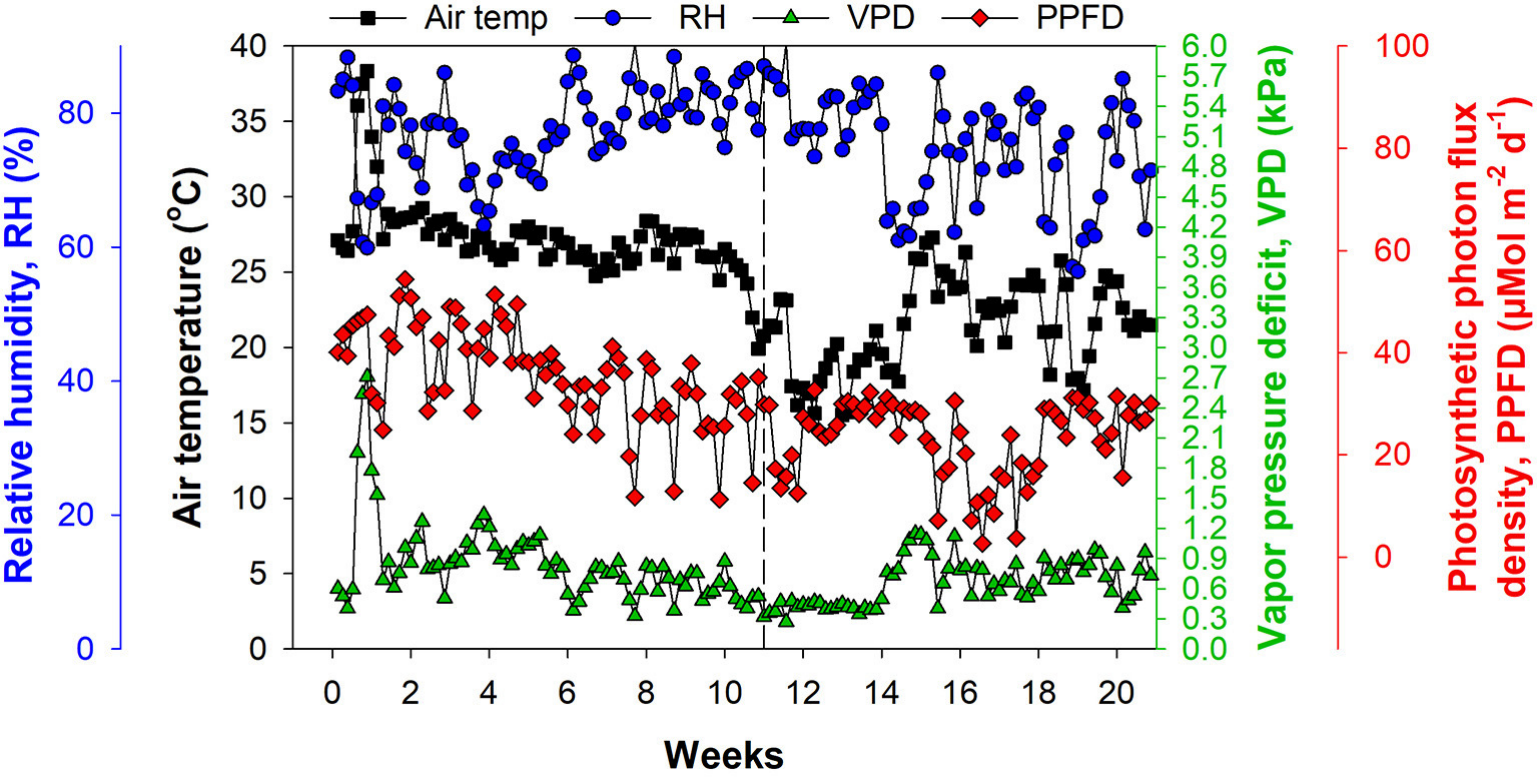
Environmental conditions inside the greenhouse with double-layer plastic roof and polycarbonate sides, equipped with a heater and pad–fan cooling system. The dashed line at Week 11 represents 12 November, 2019 and indicates the beginning of the experiment.

We used a greenhouse with a double-layer plastic roof and polycarbonate sides, equipped with a heater and pad–fan cooling system for climate control. The environmental conditions inside the greenhouse were monitored using a temperature and humidity sensor (HMP 60; Vaisala, Helsinki, Finland) and a photosynthetic photon flux density (PPFD) sensor (#3886i; Spectrum Technologies, Aurora, IL, United States). Vapor pressure deficit (VPD) was calculated from saturated and actual vapor pressure using air temperature and relative humidity (RH) data. Air temperature ranged from 15.5 to 38.5°C, RH from 56.3% to 90.5%, VPD from 0.28 to 4.72 kPa, and cumulative PPFD from 2.6 to 54.4 μMol m^2^ d^−1^ (Figure 1).

### Plant Material, Substrate, Disease Inoculation, and Growth Conditions

Despite being the youngest species of all citrus, the grapefruit is a vital citrus fruit produced for the fresh market in the United States. The “Ray Ruby” is the most prevalent variety in Florida's Indian River Citrus District and was chosen for the study due to its commercial importance.

Two-year-old “Ray Ruby” grapefruit plants grafted on sour orange (*C. aurantium*) rootstock were obtained from a commercial nursery (Brite Leaf Nursery; Lake Panasoffkee, FL, United States). Plants were transplanted to 10-cm wide × 40-cm tall pots with peat:perlite commercial substrate (80:20 v/v) (Fafard 1P; Sungro Horticulture, Agawam, MA, United States). The substrate for all pH treatments contained the initial nutrient concentrations (mg kg^−1^): N-total = 95.55 [nitrate (NO_3_)-N = 85.75 and ammonium (NH_4_)-N = 9.80)], *p* = 2.67, K = 90.76, Ca = 188.54, Mg = 77.56, S = 205.86, B = 0.11, Cu = 0.07, Fe = 0.10, Mn = 0.12, Zn = 0.01, pH = 5.90, and electrical conductivity = 1.80 dS m^−1^.

Half of the plants were manually grafted with HLB-affected budwood (confirmed positive) using real-time quantitative polymerase chain reaction (RT-qPCR) harvested from a commercial grove located in an HLB-endemic area. Grafted plants were maintained for 6 months in a greenhouse with drip irrigation and standard citrus fertilization until a positive test for HLB was confirmed.

### Treatments and Experimental Conditions

We tested two plant disease statuses [HLB-free or healthy (negative, HLB–) and HLB-affected (positive, HLB+)] and six substrate pHs (ranging from pH 4 to 9) with four replications for a total of 48 plants (24 HLB–and 24 HLB+). Substrate pH was adjusted with elemental sulfur (S) (Fisher Scientific International, Pittsburgh, DE, United States) and calcium hydroxide [Ca(OH)_2_] (Fisher Scientific International) according to a preliminary experiment (data not shown).

The substrates for pHs 4 and 5 were lowered by adding 2.30 g and 0.76 g of S per kg of substrate, respectively. Substrate pHs 7, 8, and 9 were adjusted by adding 2.30, 24.89, and 47.48 g of Ca(OH)_2_ per kg of substrate. We weighed 3 kg of substrate per pot and added the S or Ca(OH)_2_, mixing thoroughly. The plants were transplanted into new pots with mixed substrates. Each pH treatment included eight plants that were half healthy and half HLB+.

Plants were watered using an automated irrigation system using distilled water once a day (300 ml per container) and fertilized once a month with 15 g per plant of 20-20-20 water-soluble general-purpose fertilizer (Everris Inc., Geldermalsen, The Netherlands). The fertilizer composition is as follows: N 20%, P_2_O_5_ 20%, K_2_O 20%, Mg 0.05%, B 0.0125%, Cu 0.0125%, Fe 0.05%, Mn 0.025%, Mo 0.005%, and Zn 0.025%. All plants were visually monitored in the greenhouse during the experiment for HLB symptoms and tested periodically for *C*Las presence before and after severe symptoms of HLB appeared.

### Measurements

#### Ct Values and *C*Las Titer

Leaf samples were collected from each plant before treatment and 90 days after treatment (DAT). Short sections (10–15 cm) of symptomatic branches (leaves/twigs) with the attached leaves and petioles were sampled, placed into a sealable 3.78 L plastic bag (Ziploc, Bay City, MI, United States), kept refrigerated in ice, and protected from sunlight. The samples were analyzed by RT-qPCR in a commercial laboratory (Southern Gardens Diagnostic Laboratory, Clewiston, FL, United States). The processes to collect and analyze leaf samples are detailed in Phuyal et al. (2020). Samples that show cycle threshold (Ct) values ≤ 32 are considered HLB+. The bacterial titer was also quantified based on a standard curve.

#### Substrate pH

The substrate pH was measured once a week after transplanting by direct substrate measurement using a portable pH meter (HI99121; Hanna Instruments, Woonsocket, RI, United States). If the difference between the measured and target pH was over 0.5 of the target treatments, a 5-cm layer of the substrate was removed from the container. A new substrate with either elemental S or Ca(OH)_2_ was added to the surface to readjust the pH. We repeated this procedure once a week until the difference between the measured and the target pH was <0.5 (Week 11), then kept measuring the pH for 90 DAT.

#### Leaf Nutrient Concentration

Leaf samples were collected at the beginning and 90 DAT. We used 12 plants for the testing at the beginning of the trial and pooled the results together. At 90 DAT, we tested each replication. About 10–15 mature, fully expanded leaves from four quadrants of the plant were randomly collected for the leaf nutrient analysis. Samples were preserved in a cooler during the sampling period and subjected to acid washing before analysis. Samples were placed in an oven at 80°C overnight to dry, and the dried material from each plot was ground to pass a 1-mm mesh screen (Wiley Laboratory Mill Model 4 3375-E10; Thomas Scientific, Swedesboro, NJ, United States). Five grams of leaf samples were analyzed using the dry–ashing method and assessed by inductively coupled plasma atomic emission spectroscopy (ICP–AES) to determine the concentration of P, K, Ca, Mg, S, B, Cu, Fe, Mn, and Zn. Leaf N concentration was determined by macro dry combustion using an elemental analyzer (LECO CNS-2000; LECO Corporation, St. Joseph, MI, United States).

#### Leaf Gas Exchange

Photosynthetic rate, stomatal conductance, internal carbon dioxide (CO_2_) concentration, and transpiration rate were measured in mature leaves 90 DAT at midday with a portable infrared gas analyzer (LI-6400XT; LI-COR, Lincoln, NE, United States).

#### Plant Size

Plant height, stem diameter, and canopy width in two directions (W–E and N–S) of 48 plants were measured before treatment, 30 and 90 DAT. Canopy volume was calculated by using the geometric prolate spheroid equation: [4/3 π (plant height/2) (average canopy width)^2^] (Obreza and Rouse, 1993).

#### Plant Biomass

The canopy was cut at the substrate level at 90 DAT, and the shoots were divided into leaves and stems. The roots were removed from the substrate, rinsed carefully with tap water, the excess water removed, and weighed to determine root fresh weight. The leaves, stems, and roots were oven-dried at 65°C for 1 week, and the dry weight was determined.

### DNA Extraction, 16S rRNA Amplification, Gene Sequencing, and Phylogenetic Analysis

Substrate samples were collected 10–15 cm below the surface at 90 DAT. Two grams of the substrate from each plant were mixed from all 4 replications on a sterile tube and shipped for 16S rRNA sequencing (Omega Bioservices, Norcross, GA, United States).

The DNA samples were amplified by PCR using the forward CS1_515F (ACACTGACGACATGGTTCTACAGTGCCAGCMGCCGCGGTAA) and reverse CS2_806R (TACGGTAGCAGAGACTTGGTCTGGACTACHVGGGTWTCTAAT) primer sets. Each forward and reverse primers contained 5 μl, which amplifies the V3–V4 region of the 16S rRNA gene and includes adaptors for library preparation for next-generation sequencing. Samples with a final volume of 25 μl contained 12 ng of sample DNA and 12.5 μl 2 × KAPA HiFi HotStart ReadyMix (Kapa Biosystems, Wilmington, MA, United States). PCR tests were performed using the following protocol: An initial denaturation step performed at 95°C for 3 min followed by 25 cycles of denaturation (95°C, 30 s), annealing (55°C, 30 s) and extension (72°C, 30 s), and a final elongation of 5 min at 72°C. The PCR product was cleaned up from the reaction mix with Mag–Bind RxnPure Plus magnetic beads (Omega Bio–Tek, Norcross, GA, United States). A second index PCR amplification, used to incorporate barcodes and sequencing adapters into the final PCR product, was performed in 25 μl reactions using the same master mix conditions as described above. The cycling conditions were as follows: 95°C for 3 min, followed by eight cycles of 95°C for 30 s, 55°C for 30 s and 72°C for 30 s. A final, 5-min elongation step was performed at 72°C.

Twelve substrate samples were sequenced *via* Illumina (Illumina, Inc., San Diego, CA, United States) targeting the 16S ribosomal region. The Fastq files were uploaded to a commercial software platform (Geneious Prime 2020.2.1; Biomatters, San Diego, CA, United States). Each sample's set of Fastq sequences was queried utilizing the NCBI's BLAST component tool component (NCBI 2021, Baltimore, MD, United States). All sample sequence ends were trimmed for any ambiguities using the Geneious Prime default toolset. The commercial software used a multiple sequence alignment, followed by selecting the top-ranking sequences based on a bit score value more than 150.00. The sequences were then used to construct a phylogenetic tree with standard settings at a 93% similarity rate configuration setting. A Tamura–Nei distance model with neighbor-joining tree building was constructed, using a resampling bootstrap method with random seeding at the software default setting of 217,665 and replicate number at 18–25 consensus phylogenetic trees per sample (Biomatters Ltd, 2020). Each tree had an average sum of branch length value of 0.030, shown below on each of the phylogenetic trees. The evolutionary distances were computed using the Maximum Composite Likelihood method (Felsenstein, 1985) and are in the units of the number of base substitutions per site. All positions with <93% of site coverage were eliminated pairwise using alignments matrices built using an open gap value of 12 and a gap extension penalty value of 3 (partial deletion option) (Tamura et al., 2004).

### Experimental Design and Statistical Analysis

We tested two disease statuses [HLB-free or healthy (negative, HLB–) and HLB-affected (positive, HLB+)] and six pH values (4, 5, 6, 7, 8, 9) in a 2 × 6 factorial arranged on a complete randomized design with four replications. The statistical analysis was performed using RStudio (RStudio Team, 2022). A generalized linear model (GLM) analyzed error variance (ANOVA) in the main factors and their interaction. The data were checked for linear model assumptions, and transformations were carried out for significant variables. Estimated marginal means were computed when interactions were significant, and mean separation was performed using Tukey at 5% probability (*p* < 0.05). The Ct value was analyzed as a non-parametric response, with the main effects of disease status and substrate pHs; aligned rank tools (ARTools) was performed for the Ct response, as the original model presents an interaction. After the non-parametric test's non-significant interaction validation, the Kruskall–Wallis test was performed, and mean separation was performed with the Wilcoxon test. Bacterial titer was not orthogonal for ARTools; therefore, it was analyzed as a GLM, with Gaussian family and identity link. A *post-hoc* analysis was performed for multiple comparisons, adjusted for Bonferroni and using Student–Newman–Keuls (SNK) test for adjusted means at 5% probability (*p* < 0.05).

## Results

### Ct Values and *C*Las Titer

As expected, the Ct value was solely influenced by the disease status, with all plants inoculated with HLB+ buds presenting Ct values below 32; therefore, plants were positive for HLB at 0 and 90 DAT (Table 1, *p* < 0.0001). The increase of substrate pH on HLB+ plants was insufficient to suppress bacterial growth, and the Ct values were consistently below 32. At 90 DAT, Ct values increased for HLB+ plants grown in pHs 7 and 8, reaching the closest to 32 Ct values (Table 1).

**Table 1:** Ct value and *C*Las titer of “Ray Ruby” grapefruit (*C. paradisi*) on sour orange (*C. aurantium*) rootstock exposed to different HLB disease statuses [HLB-free or healthy (negative, HLB–) and HLB-affected (positive, HLB+)] under an increasing range of substrate pHs (4–9) at 0 and 90 DAT.

**Factor**	**Ct value** ^a^		***C*** **Las titer (ng DNA/100 mg tissue)**	
	**0 DAT**	**90 DAT**	**0 DAT**	**90 DAT**
**Disease status**				
HLB–	40.0 ± 0.0^a^	40.0 ± 0.0^a^	0 ± 0^b^	168 ± 167.92^b^
HLB+	30.2 ± 0.2^b^	30.2 ± 0.4^b^	3,102,750 ± 434,391^a^	17,385,792 ± 2,982,332^a^
**Substrate pHs**				
4	35.2 ± 1.8	35.0 ± 1.9	989,500 ± 480,214^b^	6,131,250 ± 2,659,456
5	34.7 ± 2.0	34.8 ± 2.0	2,912,250 ± 1,298,581^a^	10,037,500 ± 4,778,708
6	35.3 ± 1.8	34.6 ± 2.0	1,140,125 ± 513,191^b^	8,441,250 ± 3,806,543
7	35.2 ± 1.8	35.4 ± 1.9	1,133,750 ± 489,668^b^	8,483,250 ± 4,424,632
8	35.4 ± 1.8	35.9 ± 1.8	896,125 ± 408,112^b^	6,282,875 ± 5,050,989
9	34.9 ± 1.9	34.9 ± 2.0	2,227,500 ± 920,558^ab^	12,781,754 ± 7,650,152
	*p-value*			
Disease status	<0.0001^**^	<0.0001^**^	<0.0001^**^	<0.0001^**^
pH	0.0600	0.2500	0.005^**^	0.842
Disease status × pH	0.1300	0.1600	0.005^**^	0.842

a *Ct values were computed with the Kruskal–Wallis test and CLas titer as a GLM*.

*Means ± standard error (n = 4) followed by the same letters are not different by the SNK test (α = 0.05). Significance codes*:

** *0.01*;

* 0.05.

**Figure 2:**
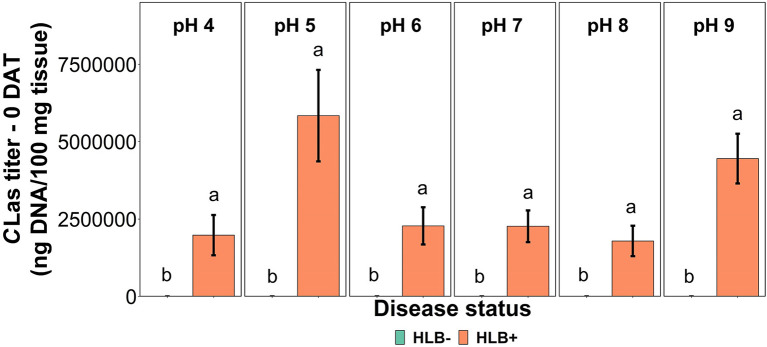
*C*Las titer in “Ray Ruby” grapefruit (*C. paradisi*) on sour orange (*C. aurantium*) rootstock exposed to different HLB disease statuses [HLB-free or healthy (negative, HLB–) and HLB-affected (positive, HLB+)] under an increasing range of substrate pHs (4–9) at 0 DAT. Means ± standard error (*n* = 4) followed by the same letters are not different by SNK test (α = 0.05) within each substrate pH for different disease status.

The *C*Las bacterial titer on leaf tissue was affected by the interaction between disease status and substrate pHs tested at the 0 DAT (Table 1 and Figure 2, *p* = 0.005). However, we compared HLB– and HLB+ plants, and the magnitude of the bacterial titer (0 compared to +3M ng DNA/100 mg tissue on 0 DAT and 168 compared to +17M ng DNA/100 mg tissue on 0 DAT, Table 1) created a statistical artifact without real biological relevance (Figure 2). The acidic and alkaline pHs 5 and 9 yielded the highest concentration of *C*Las per 100 mg of tissue (Figure 2), whereas the other substrate pHs tested were not different from each other. At 90 DAT, neither the pHs nor the interaction was significant (Table 1, *p* = 0.842).

### Substrate pH

**Figure 3:**
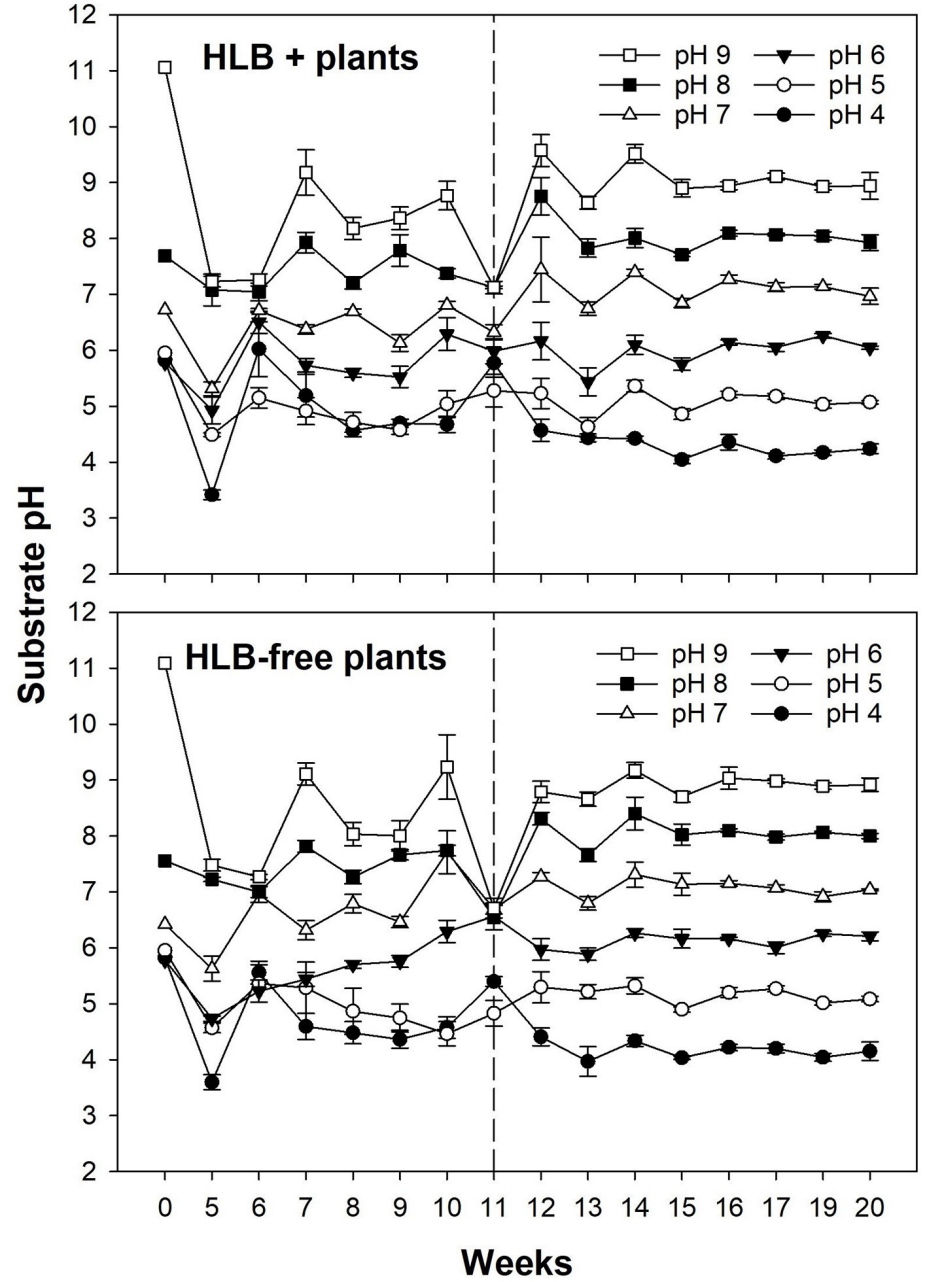
Substrate pH of plants exposed to different HLB disease statuses [HLB-free or healthy (negative, HLB–) and HLB-affected (positive, HLB+)] under an increasing range of substrate pHs (4–9). The treatments were applied for 11 weeks until the top layer of substrate started being replaced weekly, clearly stabilizing the substrate pH. The dashed line at Week 11 (12 November, 2019) indicates the beginning of the experiment. Each data point represents the mean ± standard error (*n* = 4).

The substrate pH had similar responses in both healthy and HLB+ plants before and after the treatments (Figure 3). As the pH measurements were unstable for the first 11 weeks in both healthy and HLB+ plants, a substrate with either S or Ca(OH)_2_ was added at the upper layer to maintain the pH in the targeted range.

### Leaf Nutrient Concentration

The leaf nutrient concentration at the beginning of the trial was N = 2.50%, P = 0.18%, K = 2.50%, Mg = 0.33%, Ca = 2.34%, S = 0.35%, B = 294 ppm, Cu = 298 ppm, Fe = 69 ppm, Mn = 62 ppm, and Zn = 35 ppm. B and Cu were high since the nursery constantly sprays to control pests and diseases, but grapefruit tolerates high concentrations of these elements.

The disease status and substrate pHs affected macro and micronutrients at 90 DAT differently; *p*-value was not influenced by any of the main factors or the interaction (Table 2). N and S concentrations were affected independently by disease status and substrate pHs (Table 2, *p* < 0.01). HLB+ plants acquired more N than healthy plants, whereas the S concentration in healthy plants was higher than HLB+ plants. N concentrations were lower in plants grown under pH 6 and 9 than the other pHs tested, but they were not deficient based on Morgan et al. (2021) recommendations. S leaf concentrations decreased with the increase in alkalinity of the substrate.

**Table 2:** Leaf mineral nutrient concentration of “Ray Ruby” grapefruit (*C. paradisi*) on sour orange (*C. aurantium*) rootstock exposed to different HLB disease statuses [HLB-free or healthy (negative, HLB–) and HLB-affected (positive, HLB+)] under an increasing range of substrate pHs (4–9) at 90 DAT.

	***N* (%)**	***P* (%)**	***K* (%)**	**Ca (%)**	**Mg (%)**	**S (%)**	**B (ppm)**	**Cu (ppm)**	**Fe (ppm)**	**Mn (ppm)**	**Zn (ppm)**
**Disease status**											
HLB–	2.70 ± 0.04^b^	0.155 ± 0.005	2.54 ± 0.03	2.26 ± 0.05^a^	0.31 ± 0.006^a^	0.38 ± 0.02^a^	538 ± 29^a^	84.9 ± 4.2^b^	117 ± 8	55.0 ± 3	23.1 ± 0.7
HLB+	2.89 ± 0.06^a^	0.15 ± 0.002	2.48 ± 0.04	2 ± 0.04^b^	0.27 ± 0.007^b^	0.34 ± 0.01^b^	457 ± 26^b^	98.3 ± 7.0^a^	105 ± 7	51.2 ± 3	22.1 ± 0.5
**Substrate pHs**											
4	2.99 ± 0.09^a^	0.0033 ± 0.16	2.31 ± 0.05^b^	2 ± 0.10	0.28 ± 0.02^b^	0.45 ± 0.03^a^	596 ± 60	69.8 ± 7.0^b^	113 ± 7^abc^	56.1 ± 3^ab^	22.0 ± 1.0
5	2.98 ± 0.09^a^	0.0041 ± 0.15	2.52 ± 0.04^ab^	2.16 ± 0.12	0.02 ± 0.02^ab^	0.43 ± 0.01^a^	550 ± 59	79.2 ± 3.4^ab^	107 ± 8^abc^	64.2 ± 6^a^	25.4 ± 1.0
6	2.61 ± 0.07^b^	0.0112 ± 0.17	2.48 ± 0.06^ab^	2.16 ± 0.05	0.31 ± 0.01^ab^	0.37 ± 0.01^b^	485 ± 47	113 ± 13.0^a^	152 ± 16^a^	55.9 ± 3^ab^	21.8 ± 0.9
7	2.86 ± 0.07^ab^	0.006 ± 0.16	2.5 ± 0.03^ab^	2.14 ± 0.04	0.26 ± 0.01^ab^	0.34 ± 0.01^bc^	473 ± 34	72.5 ± 2.6^b^	118 ± 12^ab^	53.2 ± 5^ab^	21.1 ± 1.0
8	2.73 ± 0.10^ab^	0.0038 ± 0.14	2.66 ± 0.03^a^	2.21 ± 0.12	0.28 ± 0.01^a^	0.31 ± 0.02^bc^	461 ± 28	102.0 ± 7.2^ab^	100 ± 9^bc^	42.2 ± 2^b^	22.8 ± 0.6
9	2.61 ± 0.06^b^	0.0046 ± 0.15	2.58 ± 0.08^a^	2.11 ± 0.07	0.3 ± 0.01^a^	0.29 ± 0.02^c^	420 ± 45	113.0 ± 10.1^a^	75 ± 4^c^	46.6 ± 4^b^	22.6 ± 0.9
Optimum level range*^y^*	2.5–2.7	0.12–0.16	1.2–1.7	3.0–4.9	0.3–0.49	0.2–0.4	36–100	5–16	60–120	25–100	25–100
	*p-value*										
Disease status	0.0013^**^	0.3300	0.2000	<0.0001^**^	<0.0001^**^	0.0035^**^	0.0359^*^	0.0370^*^	0.1300	0.2100	0.1600
pH	0.0001^**^	0.0700	0.0010^**^	0.4000	0.5600	<0.0001^**^	0.1053	<0.0001^**^	<0.0001^**^	<0.0001^**^	0.0160
Disease status × pH	0.1100	0.0600	0.8000	0.0300^*^	0.2800	0.2400	0.5041	0.0180^*^	0.5400	0.0620	0.0500^*^

*Means ± standard error (n = 4) followed by the same letters are not different by Tukey honest significant difference (HSD, α = 0.05). Significance codes*:

** *0.01*;

* *0.05*.

y *According to Morgan et al. (2021)*.

The substrate pHs tested affected K, Fe, and Mn, while Mg and B were solely influenced by the disease status (Table 2, *p* < 0.05). “Ray Ruby” plants grown in pH 4 had the lowest K leaf concentration, while more alkaline substrate pHs (8 and 9) reached the maximum concentrations. K leaf concentration fell in the high category, according to Morgan et al. (2021). Fe had a distinct concentration distribution upon the substrate pH in which the plants were grown (Table 2). Neutral substrate pH allowed more Fe in “Ray Ruby” leaves than very alkaline pHs (pH 8 and 9), ranging from high to optimal levels according to Morgan et al. (2021), respectively. Mn leaf concentration throughout the different substrate pH tested was not as variable as Fe. The substrates with slightly acidic properties yielded more Mn in leaves than the alkaline pHs tested (Table 2), but all pHs had Mn in their optimal concentration range.

Healthy plants had higher B and Mg concentrations than HLB+ plants (Table 2, *p* < 0.05). B leaf concentrations were in the excess range for healthy and HLB+ trees (Morgan et al., 2021). These high values are a residual effect from the nursery since they constantly spray to control pests and diseases. Mg concentration in healthy leaves reached the optimum range indicated by Morgan et al. (2021), while HLB+ plants were still in the low range.

**Figure 4:**
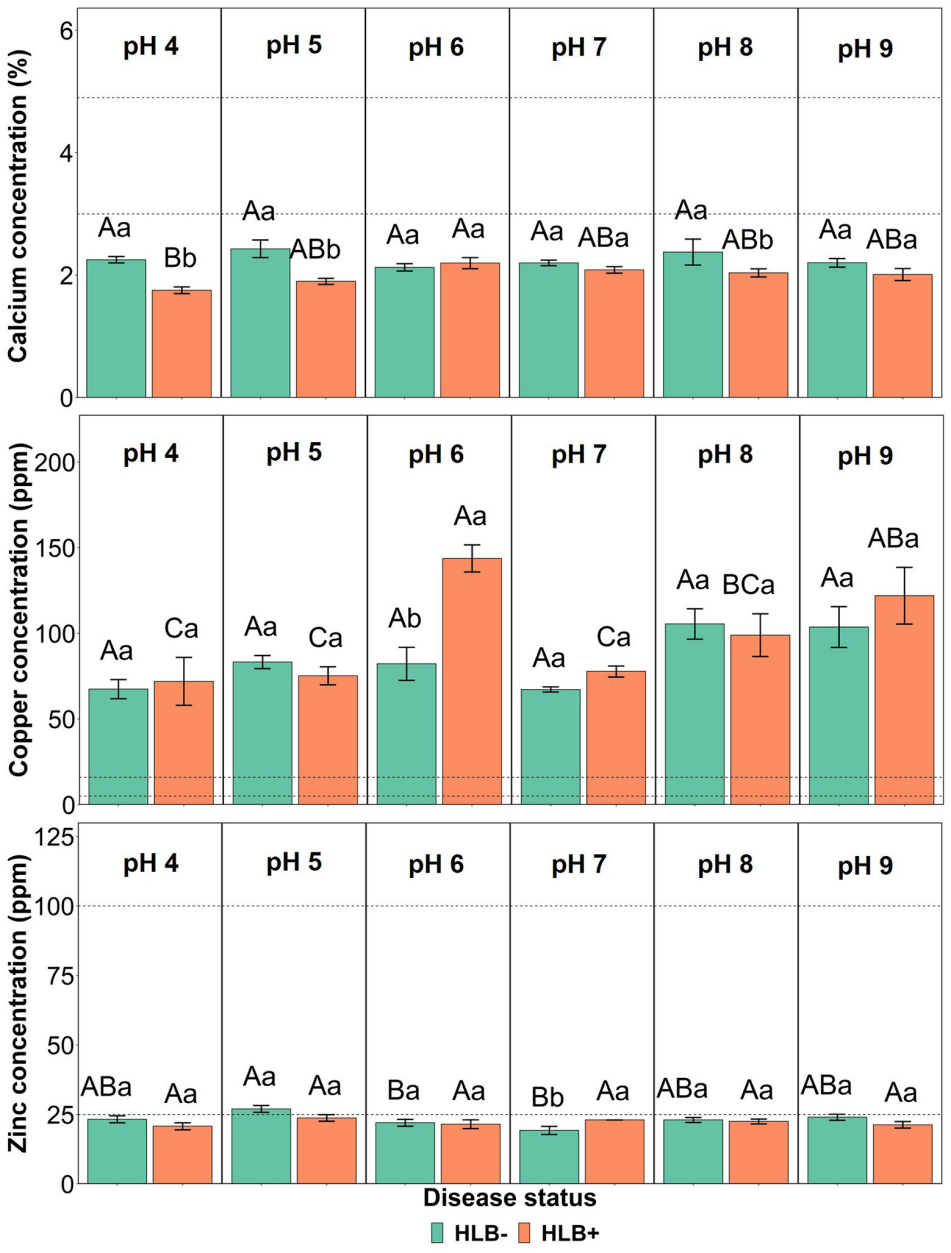
Leaf Ca, Cu, and Zn concentration interactions of “Ray Ruby” grapefruit (*C. paradisi*) on sour orange (*C. aurantium*) rootstock exposed to different HLB disease statuses [HLB-free or healthy (negative, HLB–) and HLB-affected (positive, HLB+)] under an increasing range of substrate pHs (4–9). Means ± standard error (*n* = 4) followed by the same letters are not different by Tukey honest significant difference (HSD, α = 0.05). Means with the same uppercase letters are not different on each substrate pH within disease status and means with the same lowercase letters are not different within the disease status per substrate pH. The dashed lines represent the optimum range of the element, according to Morgan et al. (2021).

The interaction between the disease status and substrate pHs influenced Ca, Cu, and Zn leaf concentration (Figure 4, *p* ≤ 0.05). The Ca concentration in healthy and HLB+ leaves of “Ray Ruby” plants grown under pHs 6, 7, and 9 was not different (Figure 4, lowercase letters), whereas healthy plants accumulated more Ca in leaves than HLB+ plants. When looking into the effect of the different pHs on Ca concentration, HLB+ plants had the highest Ca concentration under pH 6 (Figure 4, orange bars, uppercase letters), compared to very acidic pH 4. However, the treatments had Ca leaf levels lower than the optimal ranges. On the contrary, the Cu leaf concentration of HLB+ plants grown under pH 6 substrate was much higher than the minimum indicated by Fitts et al. (1967) and Colombo et al. (2014) (Figure 4, lowercase letters). Within healthy plants, all substrate pHs yielded Cu leaf concentration in the excess range, without an effect of the substrate pH (Figure 4, green bars, uppercase letters). Healthy “Ray Ruby” plants grown in pH 5 substrate were the only treatment to reach the optimal Zn concentration (25 ppm), according to Morgan et al. (2021) (Figure 4, green bars, uppercase letters). All the other 11 interactions were classified as low Zn leaf concentration, and HLB+ plants' responses to Zn accumulation upon substrate pHs were not different (Figure 4, orange bars, uppercase letters). The *C*Las presence influenced Zn accumulation in leaves only in plants grown under pH 7, as HLB+ plants had higher Zn leaf concentration than HLB– “Ray Ruby” leaves (Figure 4, lowercase letters).

### Leaf Gas Exchange

**Figure 5:**
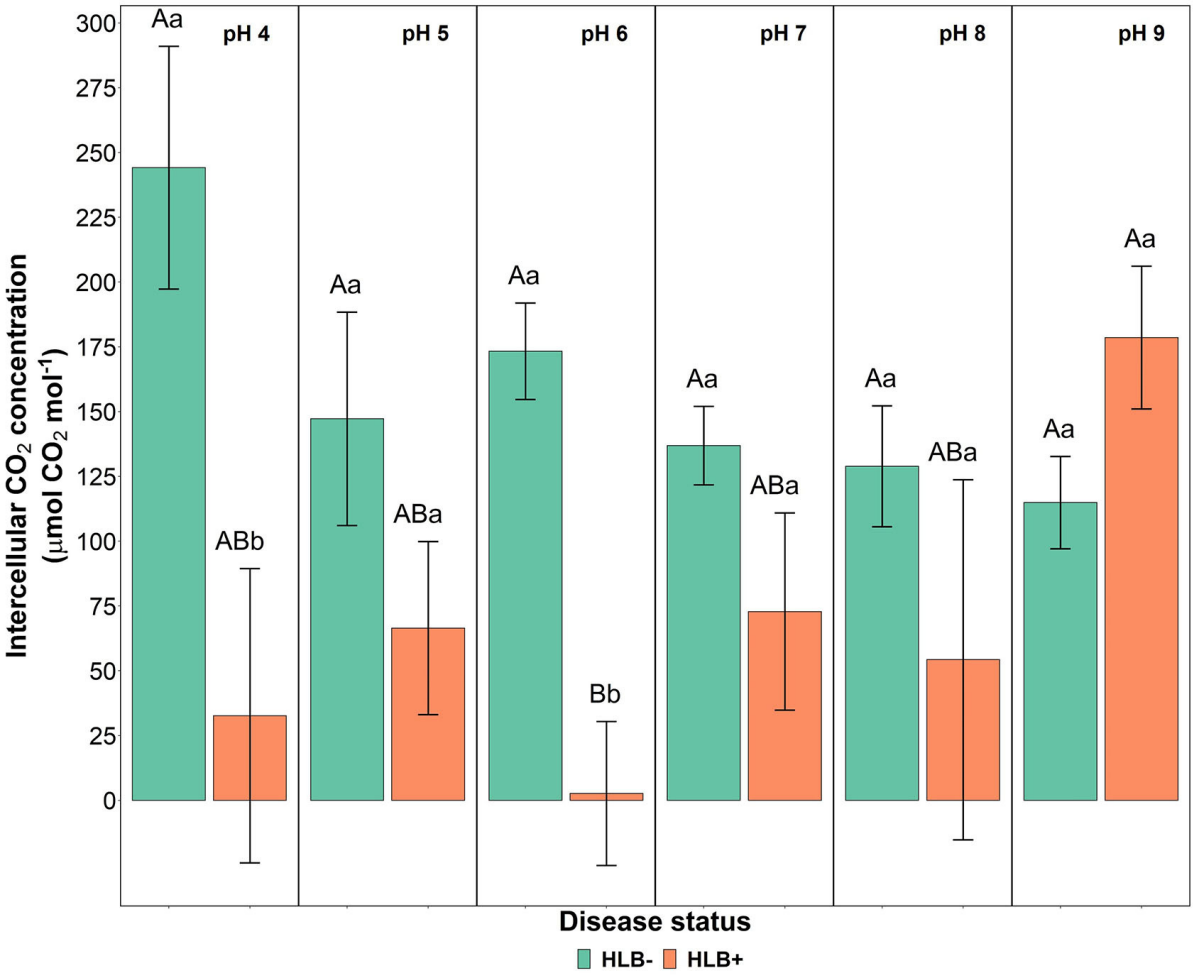
Leaf intercellular CO_2_ concentration interaction of “Ray Ruby” grapefruit (*C. paradisi*) on sour orange (*C. aurantium*) rootstock exposed to different HLB disease statuses [HLB-free or healthy (negative, HLB–) and HLB-affected (positive, HLB+)] under an increasing range of substrate pHs (4–9). Means ± standard error (*n* = 4) followed by the same letters are not different by Tukey honest significant difference (HSD, α = 0.05). Means with the same uppercase letters are not different on each substrate pH within disease status, and means with the same lowercase letters are not different within the disease status per substrate pH.

The photosynthetic rate, stomatal conductance, and the transpiration rate did not differ based on the disease statuses and substrate pHs (Table 3, *p* > 0.05). However, the internal CO_2_ concentration responded to the interaction of disease status and substrate pHs (Table 3, *p* = 0.02). HLB+ plants under pH 9 showed a higher concentration of internal CO_2_ compared to pH 6 (Figure 5, pH, uppercase letters). The internal CO_2_ was higher in healthy plants under pH 4 and 6 than HLB+ plants in the same conditions (Figure 5, pH, lowercase letters).

**Table 3:** Leaf gas exchange of “Ray Ruby” grapefruit (*C. paradisi*) on sour orange (*C. aurantium*) rootstock exposed to different HLB disease statuses [HLB-free or healthy (negative, HLB–) and HLB-affected (positive, HLB+)] under an increasing range of substrate pHs (4–9).

	**Net photosynthesis,** **A (μmol^**−2**^ s^**−1**^)**	**Stomatal conductance, g_**s**_ (mol m^**−2**^ s^**−1**^)**	**Internal CO_**2**_ concentration, Ci (μmol mol^**−1**^)**	**Transpiration rate, E (mmol m^**−2**^ s^**−1**^)**
**Disease status**				
HLB–	5.7 ± 0.5	0.4 ± 0.004	158 ± 14^a^	1.0 ± 0.1
HLB+	6.9 ± 0.4	0.04 ± 0.005	68 ± 20^b^	1.1 ± 0.1
**pH**				
4	6.0 ± 1.0	0.4 ± 0.007	138 ± 53	1.1 ± 0.2
5	6.5 ± 0.9	0.04 ± 0.008	107 ± 29	1.1 ± 0.2
6	6.4 ± 0.4	0.04 ± 0.006	88 ± 36	1 ± 0.1
7	7.1 ± 0.9	0.04 ± 0.007	105 ± 22	1.1 ± 0.2
8	5.4 ± 0.9	0.03 ± 0.007	92 ± 37	0.8 ± 0.2
9	6.4 ± 0.7	0.05 ± 0.011	147 ± 20	1.2 ± 0.3
		*p-value*		
Disease status	0.0600	0.9000	0.0002^**^	0.8000
pH	0.8000	0.8000	0.5000	0.7000
Disease status × pH	0.5000	0.4000	0.0200^*^	0.6000

*Means ± standard error (n = 4) followed by the same letters are not different by Tukey honest significant difference (HSD, α = 0.05). Significance codes*:

** *0.01*;

* *0.05*.

### Plant Size

“Ray Ruby” grapefruit growth was influenced by disease status and substrate pHs (Table 4). The disease status influenced plant height at 90 DAT (Table 4, *p* = 0.03), and as expected, healthy plants were taller than HLB+ plants at the study's end.

**Table 4:** Plant size and biomass of “Ray Ruby” grapefruit (*C. paradisi*) on sour orange (*C. aurantium*) rootstock exposed to different HLB disease statuses [HLB-free or healthy (negative, HLB–) and HLB-affected (positive, HLB+)] under an increasing range of substrate pHs (4–9) at 0, 30, and 90 DAT.

	**Height (m)**			**Stem diameter (mm)**			**Canopy volume (m** ^3^ **)**			**Dry shoot weight (g)**	**Dry root weight (g)**	**Root-to-shoot ratio**
	**0 DAT**	**30 DAT**	**90 DAT**	**0 DAT**	**30 DAT**	**90 DAT**	**0 DAT**	**30 DAT**	**90 DAT**	**90 DAT**	**90 DAT**	**90 DAT**
**Disease status**												
HLB–	136 ± 4	145 ± 4	153 ± 5^a^	16.8 ± 0.3^a^	17.6 ± 0.4^a^	17.9 ± 0.3^a^	2.2 ± 0.1^a^	3.0 ± 0.2^a^	3.7 ± 0.2^a^	204 ± 10^a^	156 ± 9^a^	0.77 ± 0.02^a^
HLB+	129 ± 4	134 ± 4	139 ± 4^b^	14.8 ± 0.4^b^	15.4 ± 0.4^b^	15.8 ± 0.4^b^	1.6 ± 0.2^b^	2.1 ± 0.3^b^	2.4 ± 0.3^b^	161.02 ± 10^b^	78 ± 8^b^	0.51 ± 0.03^b^
**pH**												
4	131 ± 8	137 ± 8	140 ± 8	16.1 ± 0.8	16.4 ± 0.8	16.7 ± 0.7	1.8 ± 0.3	2.3 ± 0.4	2.6 ± 0.5	172 ± 12	107 ± 16	0.67 ± 0.07^ab^
5	138 ± 5	146 ± 3	152 ± 3	15.1 ± 0.4	16 ± 0.5	16.3 ± 0.5	1.9 ± 0.2	2.7 ± 0.3	3.4 ± 0.3	192 ± 9	99 ± 12	0.51 ± 0.05^b^
6	132 ± 6	136 ± 6	146 ± 8	16.1 ± 0.8	17.1 ± 0.7	17.4 ± 0.7	1.9 ± 0.3	2.4 ± 0.4	3.1 ± 0.5	196 ± 21	119 ± 19	0.59 ± 0.05^ab^
7	124 ± 4	131 ± 6	137 ± 6	15.4 ± 1.1	16.4 ± 1.1	16.5 ± 1.1	1.6 ± 0.4	2.3 ± 0.6	3.1 ± 0.8	178 ± 27	112 ± 25	0.65 ± 0.05^ab^
8	138 ± 10	150 ± 11	160 ± 11	16.5 ± 0.7	17 ± 0.8	17.5 ± 0.8	2.2 ± 0.3	3.1 ± 0.5	3.7 ± 0.5	206 ± 17	153 ± 28	0.71 ± 0.08^a^
9	132 ± 7	136 ± 7	141 ± 8	15.6 ± 0.6	16.2 ± 0.7	16.6 ± 0.7	1.9 ± 0.3	2.2 ± 0.3	2.5 ± 0.4	153 ± 19	112 ± 18	0.71 ± 0.05^a^
	*p-value*											
Disease status	0.2	0.06	0.0300^**^	0.0001^**^	0.0002^**^	0.0003^**^	0.0080^**^	0.0040^**^	0.0003^**^	0.0019^*^	<0.0001^**^	<0.0001^**^
pH	0.7	0.42	0.26	0.53	0.76	0.65	0.67	0.43	0.28	0.17	0.13	0.0030^**^
Disease status × pH	0.4	0.42	0.22	0.0040^**^	0.0400^*^	0.05	0.0400^*^	0.0100^*^	0.0040^**^	0.08	0.39	0.57

*Means ± standard error (n = 4) followed by the same letters are not different by Tukey honest significant differences (HSD, α = 0.05). Significance codes*:

** *0.01*;

* *0.05*.

**Figure 6:**
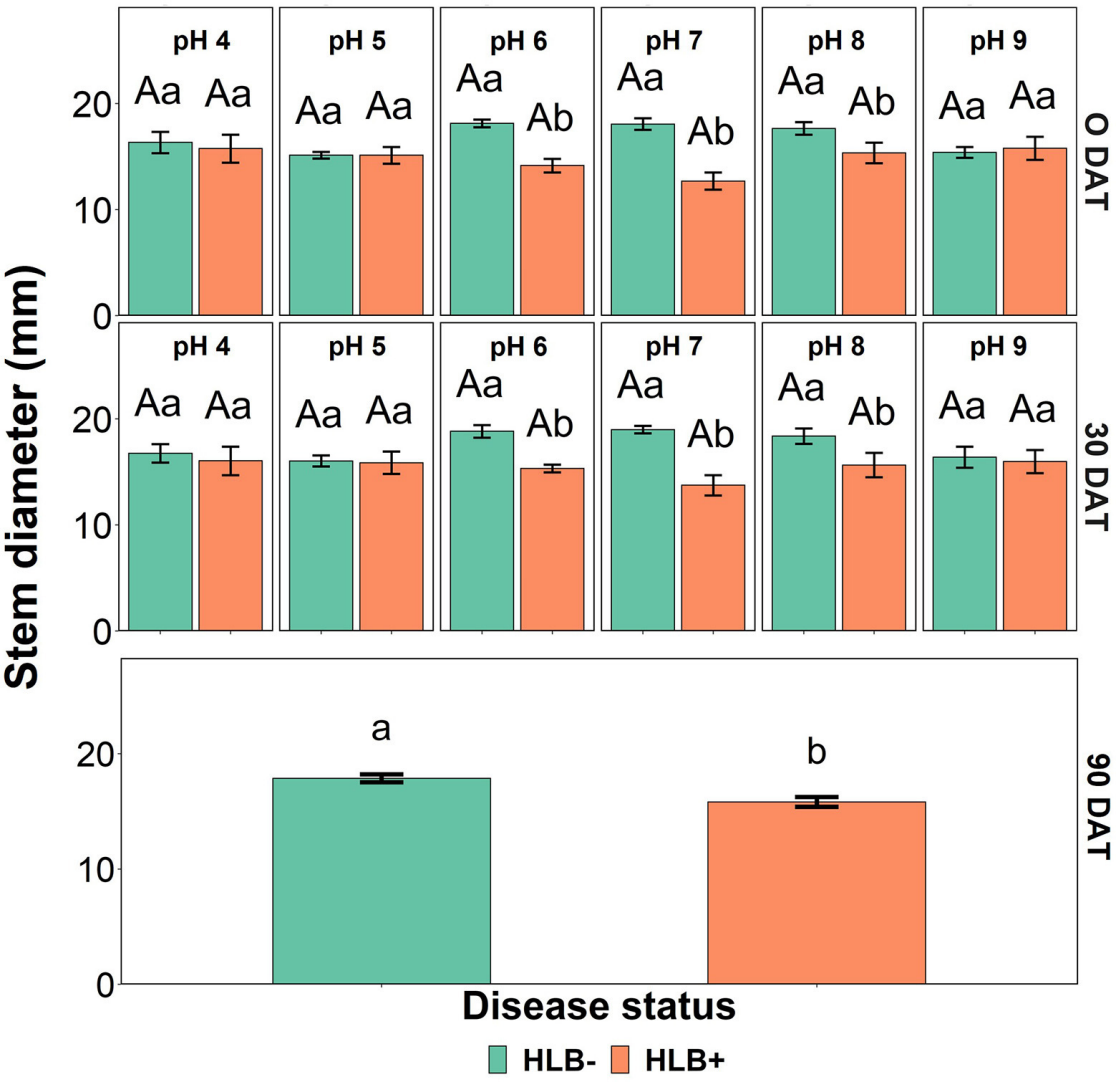
Stem diameter interactions of “Ray Ruby” grapefruit (*C. paradisi*) on sour orange (*C. aurantium*) rootstock exposed to different HLB disease statuses [HLB-free or healthy (negative, HLB–) and HLB-affected (positive, HLB+)] under an increasing range of substrate pHs (4–9) at 0, 30, and 90 DAT. Means ± standard error (*n* = 4) followed by the same letters are not different by Tukey honest significant difference (HSD, α = 0.05). Means with the same uppercase letters are not different on each substrate pH within disease status and means with the same lowercase letters are not different within the disease status per substrate pH.

The stem diameter was affected by the interaction between the disease status and the substrate pH at 0 and 30 DAT (Table 4, *p* < 0.05, and Figure 6). At 0 and 30 DAT, HLB+ plants grown in the substrate pHs 5, 7, and 8 were thinner than healthy plants. At 90 DAT, only the disease status influenced stem diameter (Table 4, *p* = 0.0003), and healthy plants were thicker than HLB+ plants.

**Figure 7:**
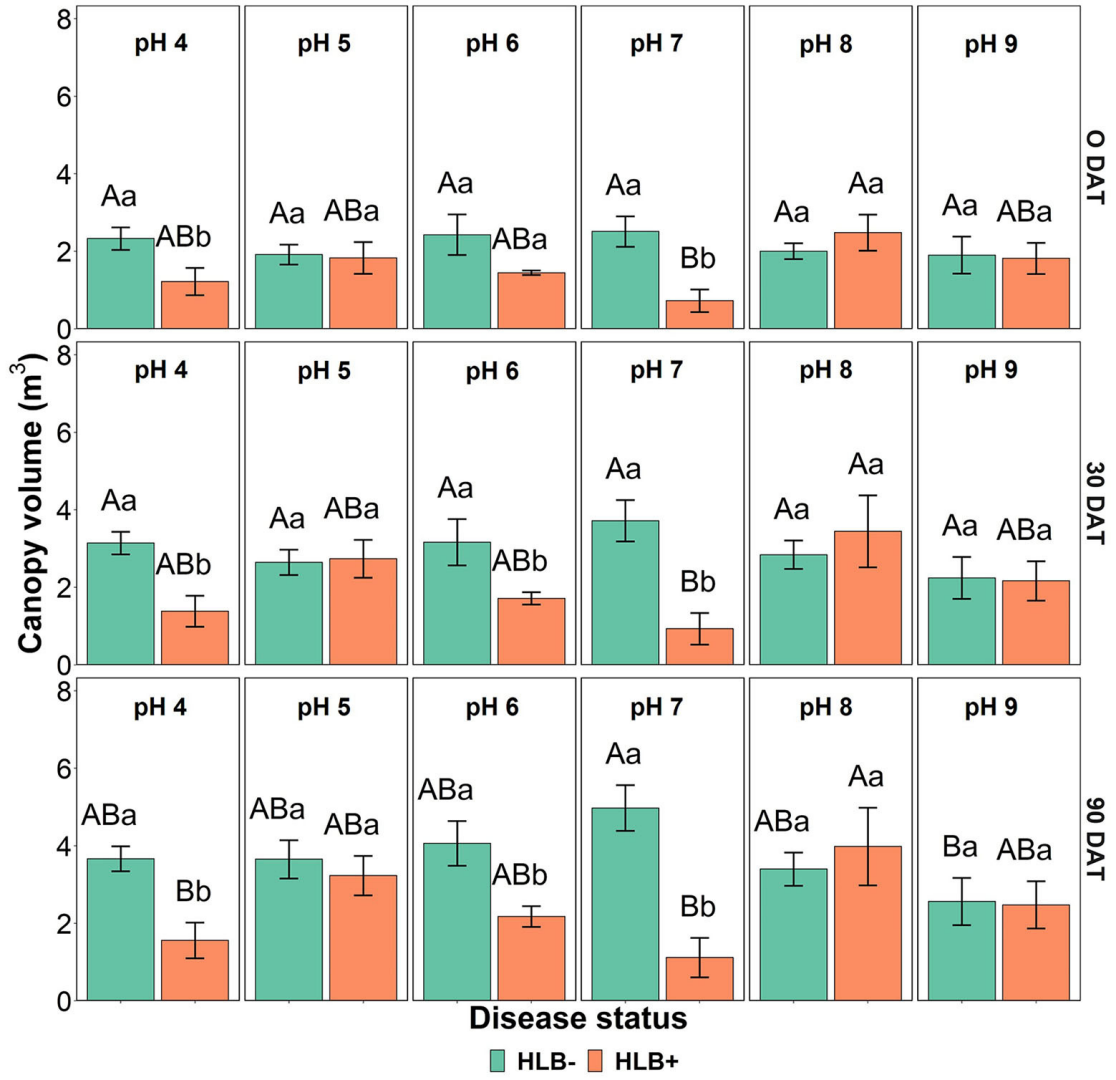
Canopy volume interactions of “Ray Ruby” grapefruit (*C, paradisi*) on sour orange (*C. aurantium*) rootstock exposed to different HLB disease statuses [HLB-free or healthy (negative, HLB–) and HLB-affected (positive, HLB+)] under an increasing range of substrate pHs (4–9) at 0, 30, and 90 DAT. Means ± standard error (*n* = 4) followed by the same letters are not different by Tukey honest significant difference (HSD, α = 0.05). Means with the same uppercase letters are not different on each substrate pH within disease status and means with the same lowercase letters are not different within the disease status per substrate pH.

The canopy volume was affected by the interaction between disease status and substrate pH (Table 4, *p* < 0.05, and Figure 7). HLB+ plants grown under pH 8 substrate had higher canopy volume than pHs 4 and 7 throughout the study (Figure 7, orange bars, uppercase letters)—although canopy volume of healthy plants grown under pH 7 was only different than substrate pH 9 at 90 DAT (Figure 7, green bars, uppercase letters).

### Plant Biomass

The dry shoot and the root weight were influenced solely by the disease status (Table 4, *p* < 0.01), while root-to-shoot ratio was influenced by both main factors (Table 4, *p* < 0.001). As expected, healthy plants accumulated more roots and shoot dry weight than HLB+ plants (Table 4), and the root-to-shoot ratio in healthy plants was 39% higher than HLB+ plants. High substrate pHs yielded a higher root-to-shoot ratio than pH 5 (Table 4).

### 16S rRNA Identification and Phylogenetic Analysis

A 16S rRNA identification analysis was conducted in different substrate pHs used to grow plants over 3 months. The primary objective of this analysis was to identify the different types of rhizospheric microbial bacteria species and their relative abundance concerning the substrate pH of healthy and HLB+ plants. Samples were collected and prepared as previously indicated for Illumina sequencing. The different substrate pHs generated 16S rRNA uncultured rhizobacterial sequences with similar kingdom taxonomic classification. Bacteria were the most detected kingdom in substrate samples from healthy and HLB+ plants (Supplementary Figures S1–S6). The substrate pHs influenced the phyla taxa, and a notable higher relative abundancy of Proteobacteria presence was detected for all substrate samples tested.

The uncultured rhizobacterial sequences from the 16S rRNA identification analysis were used methodically to construct phylogenetic trees for each of the pH treatments as described. An average of 19–25 rhizobacterial sequences were generated per healthy and HLB+ substrate samples (Supplementary Figures S1–S6). As the pH treatments changed from acid to alkaline conditions, the microbial diversity increased.

The acidic substrate conditions yielded more Proteobacteria, Acidobacteria, Actinobacteria, Firmicutes, and Verrucomicrobia phyla in healthy plants, and Planctomycetes phylum group and a relatively low presence of Acidobacteria in HLB+ samples (Table 5). Proteobacteria was the most abundant phyla seen in plants grown under acidic pH 4 substrate conditions on both disease statuses.

**Table 5:** Illumina top eight phyla classifications percentages of “Ray Ruby” grapefruit (*C. paradisi*) on sour orange (*C. aurantium*) rootstock exposed to different HLB disease statuses [HLB-free or healthy (negative, HLB–) and HLB-affected (positive, HLB+)] under an increasing range of substrate pHs (4–9).

**Phyla/Substrate pHs**	**HLB+**						**HLB–**					
	**4**	**5**	**6**	**7**	**8**	**9**	**4**	**5**	**6**	**7**	**8**	**9**
	**%**											
Proteobacteria	35.3	41.51	40.97	42.84	41.14	41.99	40.82	42.66	40.97	45.56	40.20	43.45
Firmicutes	13.5	12.21	10.81	9.51	14.31	18.64	10.76	9.70	9.85	9.93	14.30	15.20
Verrucomicrobia	11.72	8.84	5.29	5.42	7.68	4.74	10.37	10.99	4.38	5.51	6.46	5.76
Other	10	6.05	8.13	9.71	8.69	8.28	10.76	7.79	6.75	8.85	7.06	8.25
Actinobacteria	8.24	7.04	5.92	7.44	5.83	5.10	8.54	7.18	7.43	8.48	7.49	5.58
Acidobacteria	7.71	5.08	4.16	2.44	N/A	N/A	5.39	5.28	4.26	2.30	N/A	N/A
Unclassified	7	7.16	10.07	11.62	9.71	9.39	6.14	6.98	7.36	8.75	9.71	9.95
Bacteroidetes	6	7.92	10.41	7.71	8.74	7.59	6.93	5.75	8.55	8.51	7.95	7.98
Planctomycetes	3	4.18	4.24	5.77	3.90	4.27	3.28	3.67	4.4	4.41	4.43	3.83

Proteobacteria, Acidobacteria, Planctomycetes, Firmicutes, and Verrucomicrobia were the majority phyla detected on healthy and HLB+ plants grown under substrate pH 5 (Table 5). About one-fifth of pH 5 healthy and HLB+ samples indicated presence of acidophilic bacteria, influenced by the acidic conditions. In contrast, a quarter of healthy and HLB+ family classifications were grouped and clustered as “other.”

The phyla presence changed upon disease status in a lightly acidic substrate. Proteobacteria, Firmicutes, and Bacteroidetes were the most phyla detected in healthy samples grown under substrate with pH 6 (Table 5). As for HLB+ samples, Proteobacteria, Firmicutes, Bacteroidetes, and Planctomycetes were the four most abundant phyla classifications. The top three phyla detected in healthy plants grown under pH 7 included Proteobacteria, Actinobacteria, and Bacteroidetes (Table 5). For HLB+ samples, Proteobacteria, Firmicutes, and Bacteroidetes were the three most abundant phyla detected.

Samples from healthy plants grown in a slightly alkaline substrate (pH 8) had Proteobacteria, Firmicutes, and Bacteroidetes as the most detected phyla (Table 5). Moreover, Proteobacteria were the highest representative class for pH 8 for healthy and HLB+ samples, followed by Rhizobiales, Baccillales, and Sphingobacteriales. Proteobacteria was also the main phyla detected in the alkaline pH 9 substrate in healthy and HLB+ conditions, followed by Firmicutes, and Bacteroidetes (Table 5).

## Discussion

### Ct Values and *C*Las Titer

The soil and environmental conditions affect plant vigor, productivity, and disease resistance (Larkin, 2015). Previous reports have shown that multiple plant diseases caused by fungal, bacterial, viral, and oomycete agents are closely related to poor soil conditions and influenced by variations in soil pH (Holland et al., 2018; Bennett and Klironomos, 2019). The regulation of soil acidification by adding lime and increasing soil pH is beneficial for *C*Las multiplication in HLB+ plants over months of infection and inhibiting the spread of HLB disease to healthy trees (Li et al., 2020). In this study, pH influenced the *C*Las copy number in the leaf tissue at the beginning of the experiment (*p* < 0.05), as substrate pHs 5 and 9 had higher *C*Las titers than the other substrate pH tested. However, after 90 days, there was no difference amongst the substrate pHs tested (*p* = 0.842).

### Leaf Nutrient Concentration

Generally, fewer metal ions can bind to the soil colloids at low pHs, being more available for plant uptake (Jones and Jacobsen, 2005). In our study, lower substrate pHs (4 and 5) increased the concentration of N, S, and Mn in leaves, while K^+^ had a higher concentration under a slightly alkaline substrate. NO${}_{3}^{-}$ is best uptaken by the plants in an acidic condition, and its absorption process is intimately related to H^+^ and OH^−^. In acidic environments, S concentration increases when the soil contains aluminum oxides and hydrous iron (Alam et al., 1999) but also due to the replacement of the OH^−^ in the soil colloids neutralized by the presence of the H^+^ originated from the hydrolysis of Al, caused by the replacement by the cations added with the sulfate in the soil. Mn presence in leaves under neutral/alkaline pH is coherent with other soybean studies, as Mn availability is inversely related to soil pH (Monk, 1966). An increase to pH 7 or 8 speeds the oxidation of Mn^2+^ to lesser soluble forms. As confirmed in this study, a slightly acidic environment (pH 5 to 5.5) increased the Mn uptake. S concentrations were higher under low pHs as expected since the pH of the substrate was lowered with elemental S.

K^+^ presence in lower substrate pHs is rare, as H^+^ and Al^3+^ replace the cation, according to our findings. Likewise, Fe^3+^ is highly available under acidic conditions, promoting the mobilization of Fe minerals (Colombo et al., 2014). Alkaline substrate/soil pH increases root apoplast pH, in which bicarbonate neutralizes the protons pumped out of the cytosol by a Fe transporter, hampering and even blocking the Fe^3+^ reduction (Mengel, 1994).

N, Mg, S, and B leaf concentrations differed between healthy and HLB+ plants regardless of the pHs tested. A study has shown that HLB+ trees had higher N and Zn concentrations than healthy trees (Razi et al., 2011), supporting the findings of this study, as leaf N in HLB+ plants was higher than in healthy “Ray Ruby” grapefruit plants (Table 2). Mg is the central mineral element in the chlorophyll molecule composition, and it is key to several other defense mechanisms in plants (Verbruggen and Hermans, 2013). As phloem loading and unloading process is reduced by the deposition of callose and p-protein in the sieve pore of HLB+ plants (Koh et al., 2012), it is expected to have a reduction of chlorophyll synthesis; therefore, reducing Mg uptake by HLB+ plants.

Although supplied with standard fertigation solution, B leaf concentrations in healthy and HLB+ plants exceeded the maximum 100 ppm set by Morgan et al. (2021) for citrus production. This is a consequence of the nursery's nutrient management practices since they constantly spray foliar fertilizers containing B in their formulation. The few studies that compared leaf B concentration between healthy and HLB+ plants showed no difference for the disease status, with the tendency of having higher nutrient uptake efficiency in HLB+ plants (Cimò et al., 2013; Shahzad et al., 2020).

Ca, Cu, and Zn leaf concentrations responded to disease status and the substrate pHs. Although no treatment reached minimum values for optimal Ca concentration in leaves, HLB+ plants had lower Ca leaf concentration compared to healthy leaves when under pH 4, 5, and 8. Ghimire et al. (2020) found that HLB+ trees under pH 8 were deficient in Ca and Zn, corroborating the results of this study. Additionally, Shahzad et al. (2020) showed that Ca leaf concentration was higher on healthy citrus trees than HLB symptomatic leaves under the same neutral pH 7 media, while Fe leaf concentration was higher in HLB+ plants under pH 6 and pH 8 media. Our study shows the same response of “Ray Ruby” grapefruit in terms of Ca and Fe leaf concentration, as healthy plants showed the highest Ca concentration under pH 8 substrate compared to HLB+ plants under the same pH condition (Figure 4), and plants grown under pH 6 had excessive amounts of Fe in leaves (Table 2). Ca is crucial for plant growth, as part of cell wall constituents, and moreover, as signal for plant defense (Hepler, 2005). HLB+ trees are known to have stunt growth and slow recovery from ACP feeding, and this is due partially to the low acquisition and mobilization of Ca through the feeder roots to supply membrane stability (Spann and Schumann, 2009).

Ghimire et al. (2020) showed that leaf Cu concentrations were higher in HLB+ trees under pH 6 compared to pHs 7 and 8. It is widely known that HLB+ trees have lower Cu concentrations due to the limited photosynthetic activity and chlorophyll formation (Nwugo et al., 2013; Inoue et al., 2020). However, the application of Cu as a foliar spray by the nursery before the trial makes it difficult to compare between this study's findings and the literature.

The role of Zn in HLB+ trees is well-documented, as HLB symptoms often resemble Zn deficiency (Bové, 2006; Nwugo et al., 2013). Interestingly, healthy “Ray Ruby” plants grown in pH 7 substrate accumulated more Zn in leaves compared to HLB+ (*p* < 0.05) due to the improved plant nutrition and promotion of root growth (Shahzad et al., 2020). However, only healthy plants under pH 5 reached Zn optimal levels in the leaves, since alkaline pH can lead to reduced concentrations of Zn and other micronutrients (Boswell et al., 1989).

### Leaf Gas Exchange

Previous studies focused on the effect of abiotic stresses and nutrition of HLB-affected citrus trees (Romero-Conde et al., 2014; Aparicio-Durán et al., 2021), but little is known about the gas exchange response of HLB+ plants to different substrate pHs. In this study, neither the disease status nor the substrate pHs affected “Ray Ruby” net photosynthesis (Table 3). Healthy plants did not have higher photosynthesis across the substrate pHs tested (Figure 4, *p* > 0.05); however, acidic conditions yielded the highest intercellular CO_2_ concentration, as observed by Long et al. (2017). Our study shows that alkaline pH influenced the increase in intercellular CO_2_ concentration in HLB+ “Ray Ruby” plants compared to acidic/neutral substrate pH conditions, which is uncommon (Liu and Shi, 2010; Yang et al., 2011; Ghimire et al., 2020). The changes in intercellular CO_2_ are caused due to soil/substrate salinity and its relationship to citrus physiological responses (Aparicio-Durán et al., 2021). In this study, pH affected nutrient availability and possibly its solubility in the soil. Neither Na^+^ nor Cl^−^ ions were quantified, and it is not clear if salinity or the lack of salinity caused the increase in intercellular CO_2_ in HLB+ plants under alkaline substrate pH.

### Plant Size and Biomass

The disease status influenced the growth and biomass of “Ray Ruby” grapefruit plants. That is expected since the carbohydrate supply is reduced by the phloem plugging when the bacteria infect the plant, and the carbohydrate partition and usage in new leaves/shoots are jeopardized (Cimò et al., 2013). Plant height was influenced by the presence of the *C*Las at the end of the experiment (Table 4). In contrast, stem diameter was influenced by the interaction between disease status and substrate pHs at 30 DAT (Figure 6). HLB+ plants had a thinner trunk than healthy plants. However, this data differs from Ghimire et al. (2020), in which different irrigation water pHs did not affect sweet orange trunk diameter.

Canopy volume was smaller in HLB+ plants under pH 4 (acidic) and pHs 6 and 7 (neutral), as shown in Figure 7. Morgan and Graham (2019); Ghimire et al. (2020), and Yang et al. (2020) indicated that field-established HLB+ sweet orange trees might require a slightly acidic pH substrate for adequate canopy growth. However, in this study, HLB+ plants under pH 5 had a similar canopy volume as healthy plants. This result was a surprise since substrate acidification by S did not influence the canopy volume of “Ray Ruby” grapefruit plants.

As expected, root growth and biomass accumulation were higher in healthy plants compared to HLB+ plants. Root loss in HLB+ trees is well-known (Johnson et al., 2014), accounting for decreased water and nutrient uptake to support shoot development. The root-to-shoot ratio reflects the growth and dry matter accumulation between root and shoot (Lloret et al., 1999), as root growth is highly dependable on the metabolism and dry matter accumulation in the shoot. Thus, the root-to-shoot ratio tends to decrease with the increase in plant size (Monk, 1966). A meager root-to-shoot ratio also indicates poor root growth (Zhang, 1995), as noticeable in HLB+ “Ray Ruby” grapefruit tested in this study. Genetic differences between sweet oranges and grapefruit varieties could justify the opposite root-to-shoot ratio values. Unlike “Valencia” and “Midsweet” sweet oranges (Morgan and Graham, 2019; Ghimire et al., 2020), alkaline substrate conditions increased the root-to-shoot ratio in “Ray Ruby” grapefruit. Additionally, it reveals which substrate pH is suitable for optimal root growth under *C*Las infection.

### 16S rRNA Identification and Phylogenetic Analysis

There were 119 rhizobacterial species identified by the 16S rRNA gene sequencing in HLB– samples and 123 in HLB+ samples. The selected phyla represented (Planctomycetes for low pHs and Bacteroidetes for high pHs) are critical for their biological functions.

The root microbiome cluster analysis of HLB+ plants resulted in relevant differences compared to healthy plants (Table 5). The 16S rRNA gene sequencing analysis for the different pH values provided the rhizobacterial populations with acidic, neutral, and basic substrate conditions, highlighting the microorganism viability, proliferation, and potential biochemical reactions necessary for nutrient availability. Substrate samples of healthy and HLB+ plants had Acidobacteria phyla for plants grown under acidic conditions of pHs 4 and 5. The Planctomycetes phylum is prominent in all HLB– and HLB+ substrates sampled. Planctomycetacae species are a primitive gram-negative bacterium with distinct morphological and reproductive characteristics such as the lack of a peptidoglycan cell membrane layer and specific mitotic processes by dividing by polar budding instead of binary fusion (Fuerst and Sagulenko, 2011). This phylum is commonly known for its role in plant growth, e.g., oxidizing ammonium for N fixation since the process of denitrification is a critical growth function in the rhizosphere and allows for augmented nutrient availability (Henry et al., 2008). However, the phylogenetic analysis for substrates with pHs 4 and 5 in HLB+ plants (see supplementary Charts) resulted in the absence of the *Planctopirius limnophila*, a species of the Planctomycetes phyla (Fuerst and Sagulenko, 2011). In addition, metagenomic analysis from bioreactor mixed communities of *Candidatus Kuenemia stuttgartiensis*, a Planctomycetes species identified for genes encoding for oxidoreductases. Oxidoreductases are electron acceptors that can reduce Fe and Mn, two common elements in citrus plants. It is also worth noting that Planctomycetes, Verrucomicrobia, and Chlamydia (PVC) are closely related superphylum clade. These Verrucomicrobia phyla are present in all substrate HLB– and HLB+ samples with pH > 6 (Fuerst and Sagulenko, 2011).

The neutral and basic substrate samples had a higher abundance of the Bacteroidetes phylum. Bacteroidetes are gram-negative, bacillus-shaped, anaerobic bacteria. The presence of the Bacteroidetes phylum in the 16S rRNA gene sequencing analysis supports/correlates to previous studies that identified this phylum in a greenhouse and unexploited soils (Kim et al., 2006) with a relatively neutral to basic pH (Lauber et al., 2009). The most studied functionality of the Bacteroidetes is the degradation of larger complex molecules such as polysaccharides and proteins, probably indicating that more bacteria from this phylum might result in higher nutrient availability for uptake by the plants. The Bacteroidetes species are abundant in citrus trees (Xu et al., 2018), as indicated in the phyla classification charts. Furthermore, Bacteroidetes positively correlate with higher quantities at neutral to alkaline substrates, which is shown in the phyla classification charts except for a higher percentage value for the substrate pH 4 from HLB+ plants (Thomas et al., 2011). Healthy and HLB+ plants in neutral and basic substrate pHs (6, 7, 8, and 9) exhibited more microbial diversity than acid substrate pHs (4 and 5) (Supplementary Figures S1–S6). Although the 16S rRNA sequences provide the difference between the rhizobacterial microbiome across several pH treatments, little knowledge is available about the exact role or functions of the rhizobacterial microbiome and their interactions with nutrient availability and transport function. That info can be used to identify microorganisms that can improve citrus health and productivity.

## Conclusions

This study investigated the effect of a wide range of pH values on plant size and biomass, nutrient concentration, leaf gas exchange, and rhizobacterial microbiome of “Ray Ruby” grapefruit affected by HLB. The substrate pHs heavily influenced the plant size and biomass of HLB+ plants. High pH affected nutrient availability for root uptake, changing the nutrient balance throughout the plant system. As plant photosynthesis was not affected by the substrate pHs, we can indicate that pH does not recover HLB+ plants to the photosynthetic levels of healthy plants—even though high substrate pHs positively influenced internal CO_2_. No effect of substrate pH on plant disease status was induced by enhanced nutrient uptake. On the other hand, the phylogenetic analysis indicated that HLB increases the rhizobacterial population in grapefruit. The 16S rRNA gene sequencing analysis for low substrate pHs also revealed an absence of the Planctomycetes phylum in HLB+ samples. The lack of N-fixating bacteria can potentially negatively affect increased leaf N concentrations. More studies are needed to discover how specific groups of bacteria such as Planctomycetes and Bacteroidetes influence rhizospheric biochemical reactions in the rhizosphere regarding plant-disease interactions.

## Data Availability Statement

The data presented in the study are deposited in the GenBank repository, accession numbers OM836202 - OM836404.

## Author Contributions

RF and XL designed and performed experiments. FT, XL, AG, HH, XW, J-HH, and GF analyzed data. RF provided reagents and equipment. RF, FT, and XL wrote the bulk of the manuscript, with significant contributions from AG, HH, XW, J-HH, and GF. All authors contributed to the article and approved the submitted version.

## Funding

Funding for this study was provided by UF/IFAS, Fujian Special Fund for Scientific Research Institutes in the Public Interest (2020R10280017), Collaborative Innovation Project from PGFP & CAAS (XTCXGC2021006), the Science and Technology Innovation Team of FAAS (CXTD2021003-3), and Research Project of FAAS (A2017-1).

## Conflict of Interest

The authors declare that the research was conducted in the absence of any commercial or financial relationships that could be construed as a potential conflict of interest.

## Publisher's Note

All claims expressed in this article are solely those of the authors and do not necessarily represent those of their affiliated organizations, or those of the publisher, the editors and the reviewers. Any product that may be evaluated in this article, or claim that may be made by its manufacturer, is not guaranteed or endorsed by the publisher.
