# Supplementary material for: Substrate pH Influences the Nutrient Absorption and Rhizosphere Microbiome of Huanglongbing-Affected Grapefruit Plants
Source: Front Plant Sci. 2022 May 13;13:856937. doi: 10.3389/fpls.2022.856937 (PMC9141052; doi:10.3389/fpls.2022.856937)
Supplement: Supplementary file 1 [file Data_Sheet_1.docx]

**Supplemental Materials**

**
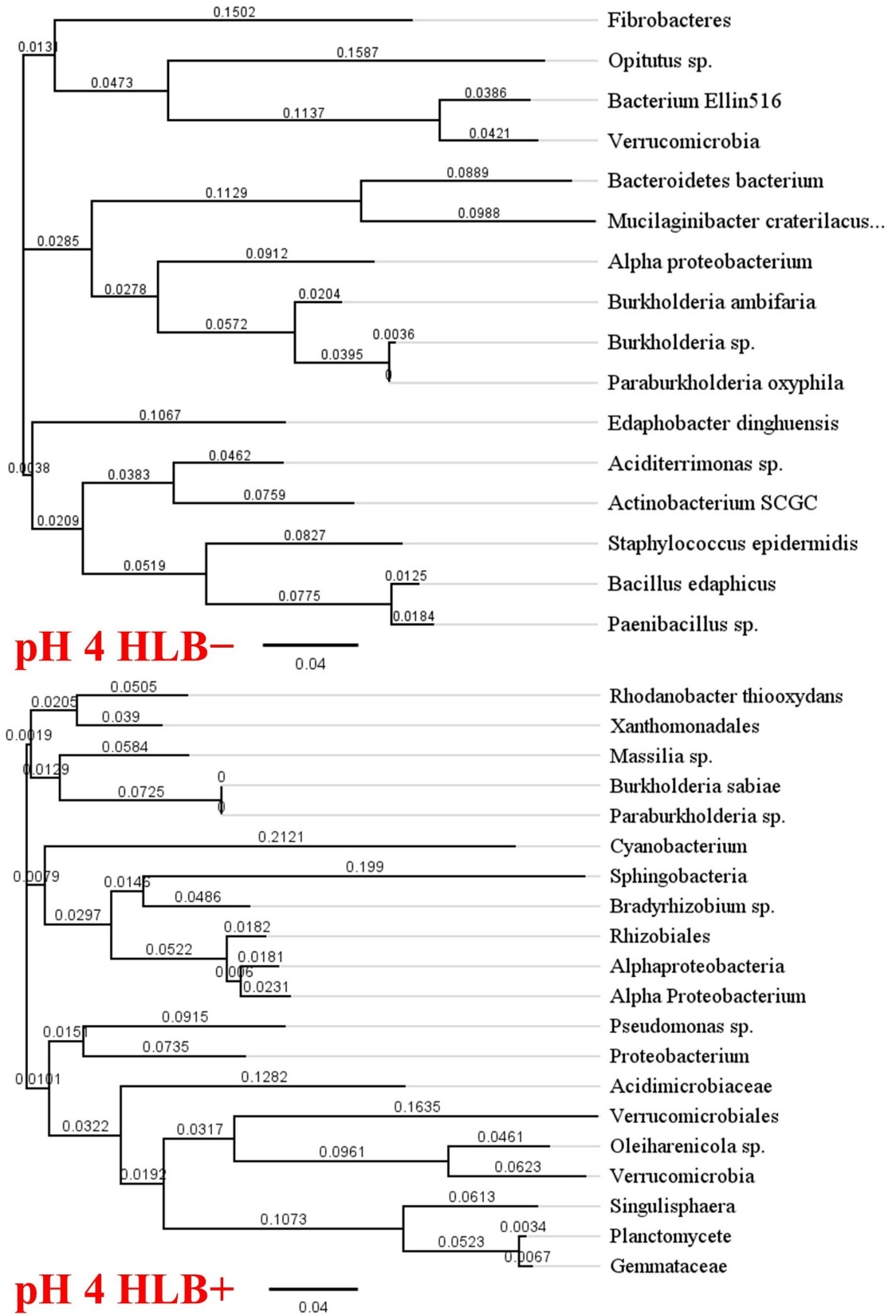
**

**Figure S1**. Bacteria phylogenetic analysis of soilless substrate under treatment pH 4 based on root microbe 16S rRNA sequencing of HLB− (top) and HLB+ (bottom) plants of ‘Ray Ruby’ grapefruit (*Citrus paradisi*) on Sour orange (*Citrus aurantium*) rootstock. The phylogenetic tree was built utilizing the neighbor-joining analysis method with bootstrap support at the nodes (Saitou & Nei, 1987).

**
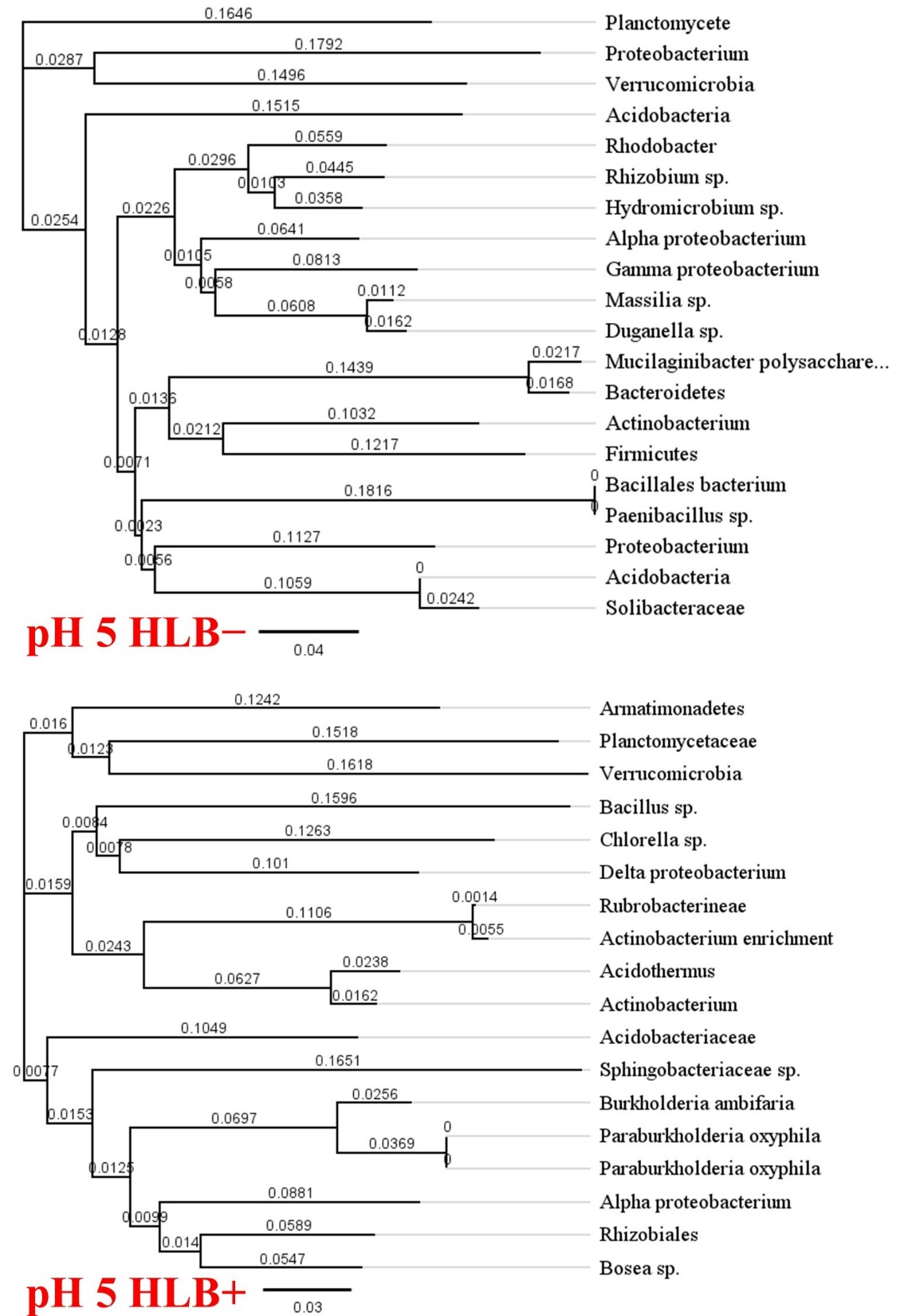
**

**Figure S2**. Bacteria phylogenetic analysis of soilless substrate under treatment pH 5 based on root microbe 16S rRNA sequencing of HLB− (top) and HLB+ (bottom) plants of ‘Ray Ruby’ grapefruit (*Citrus paradisi*) on Sour orange (*Citrus aurantium*) rootstock. The phylogenetic tree was built utilizing the neighbor-joining analysis method with bootstrap support at the nodes (Saitou & Nei, 1987).


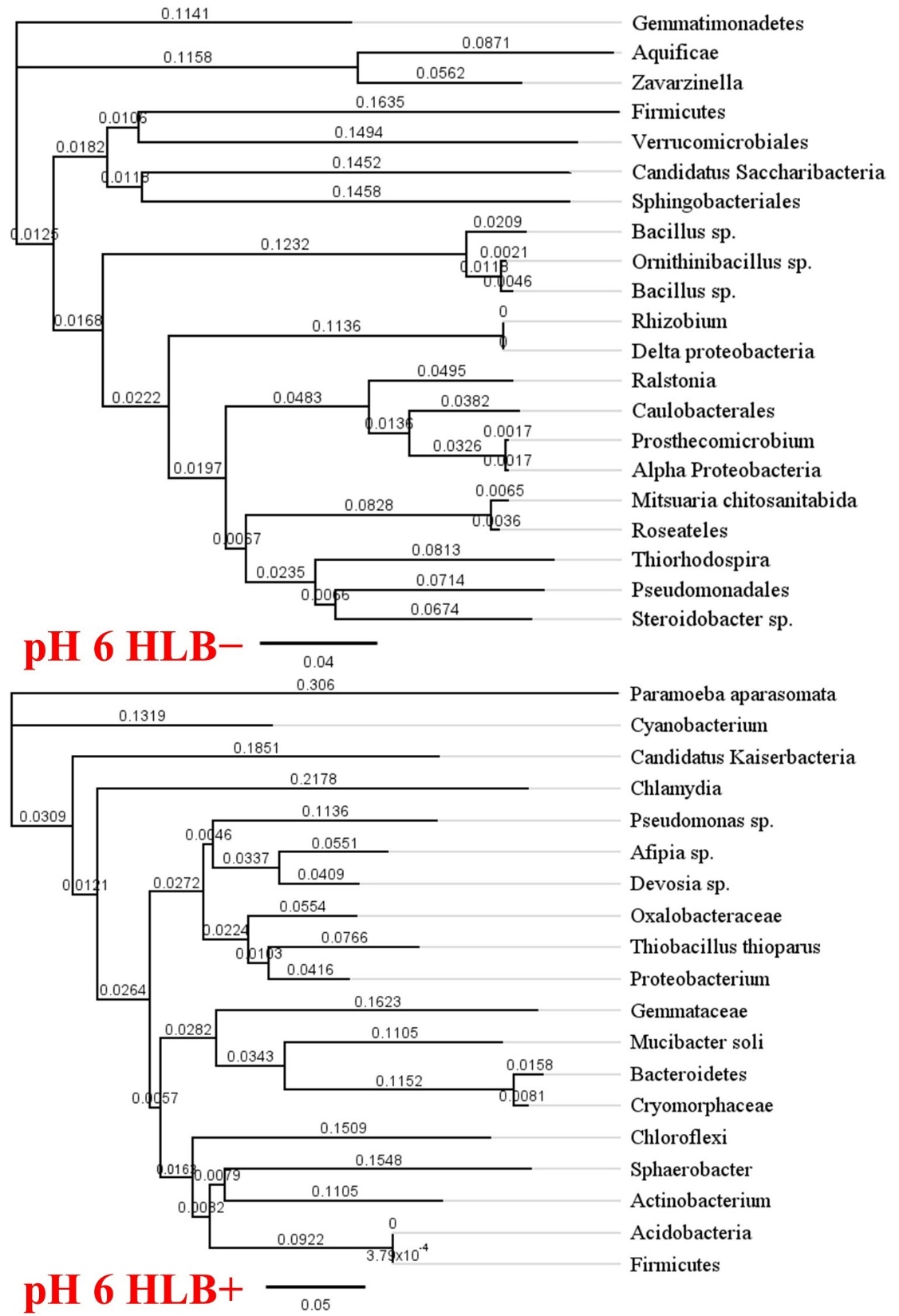


**Figure S3**. Bacteria phylogenetic analysis of soilless substrate under treatment pH 6 based on root microbe 16S rRNA sequencing of HLB− (top) and HLB+ (bottom) plants of ‘Ray Ruby’ grapefruit (*Citrus paradisi*) on Sour orange (*Citrus aurantium*) rootstock. The phylogenetic tree was built utilizing the neighbor-joining analysis method with bootstrap support at the nodes (Saitou & Nei, 1987).


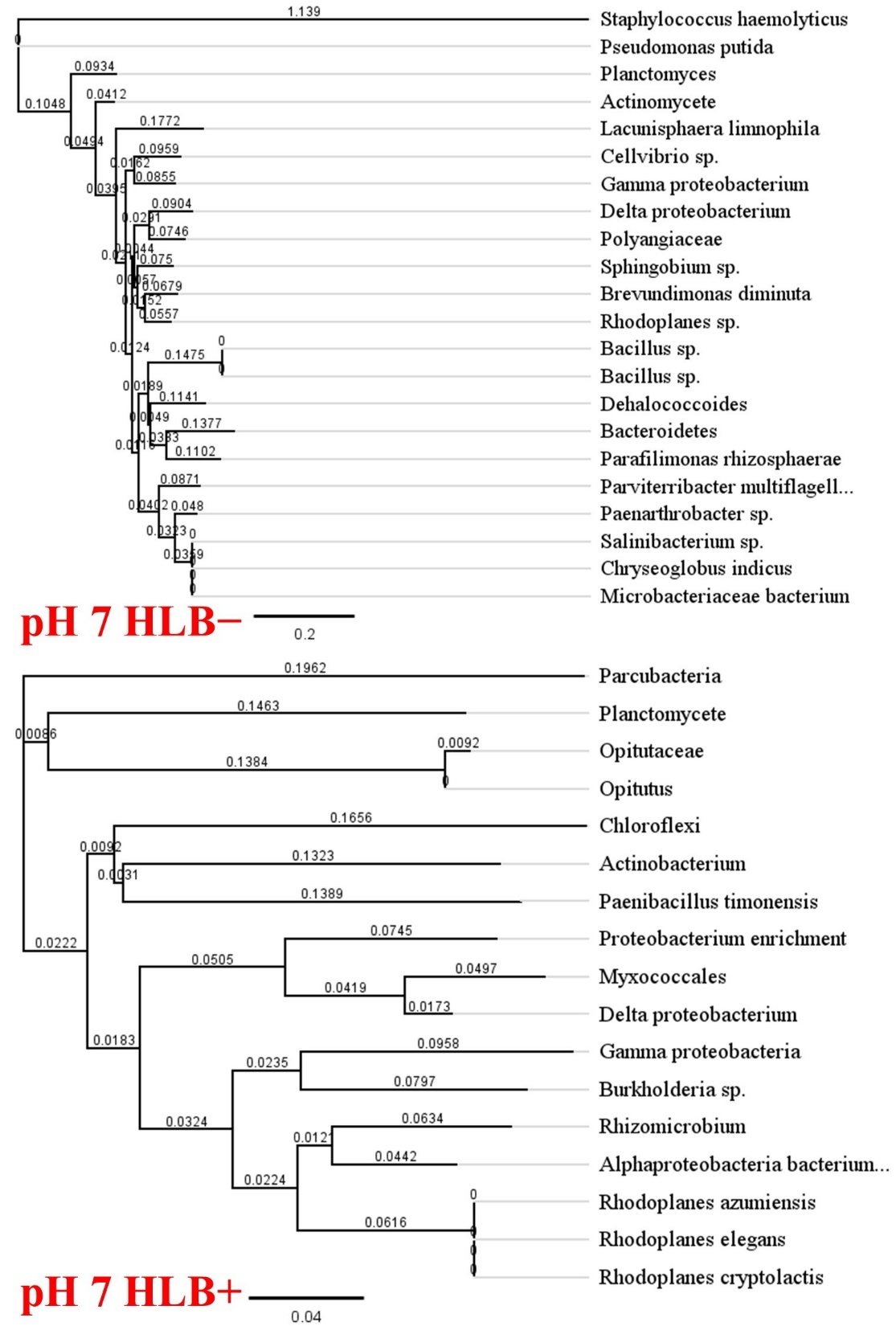


**Figure S4**. Bacteria phylogenetic analysis of soilless substrate under treatment pH 7 based on root microbe 16S rRNA sequencing of HLB− (top) and HLB+ (bottom) plants of ‘Ray Ruby’ grapefruit (*Citrus paradisi*) on Sour orange (*Citrus aurantium*) rootstock. The phylogenetic tree was built utilizing the neighbor-joining analysis method with bootstrap support at the nodes (Saitou & Nei, 1987).


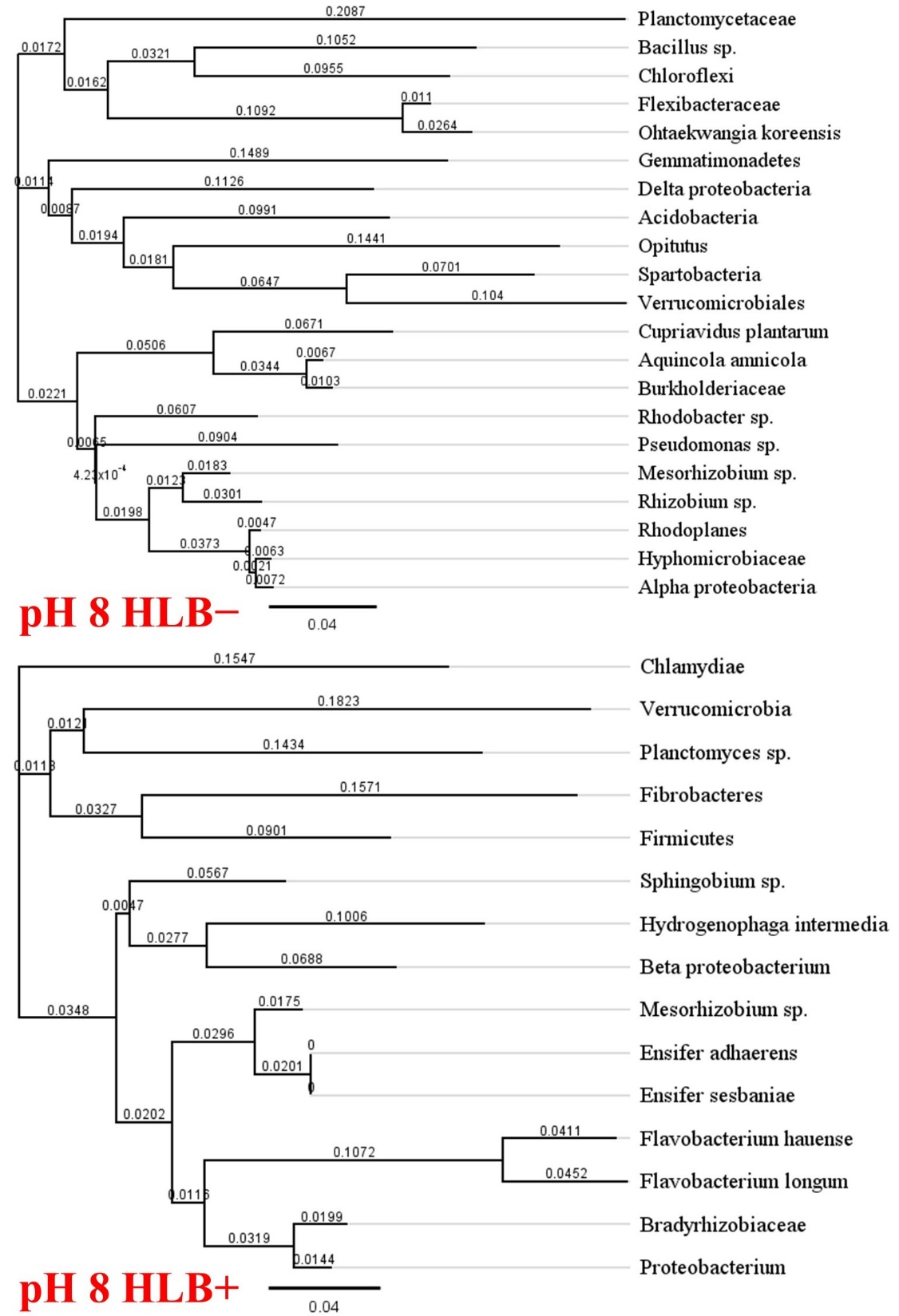


**Figure S5**. Bacteria phylogenetic analysis of soilless substrate under treatment pH 8 based on root microbe 16S rRNA sequencing of HLB− (top) and HLB+ (bottom) plants of ‘Ray Ruby’ grapefruit (*Citrus paradisi*) on Sour orange (*Citrus aurantium*) rootstock. The phylogenetic tree was built utilizing the neighbor-joining analysis method with bootstrap support at the nodes (Saitou & Nei, 1987).


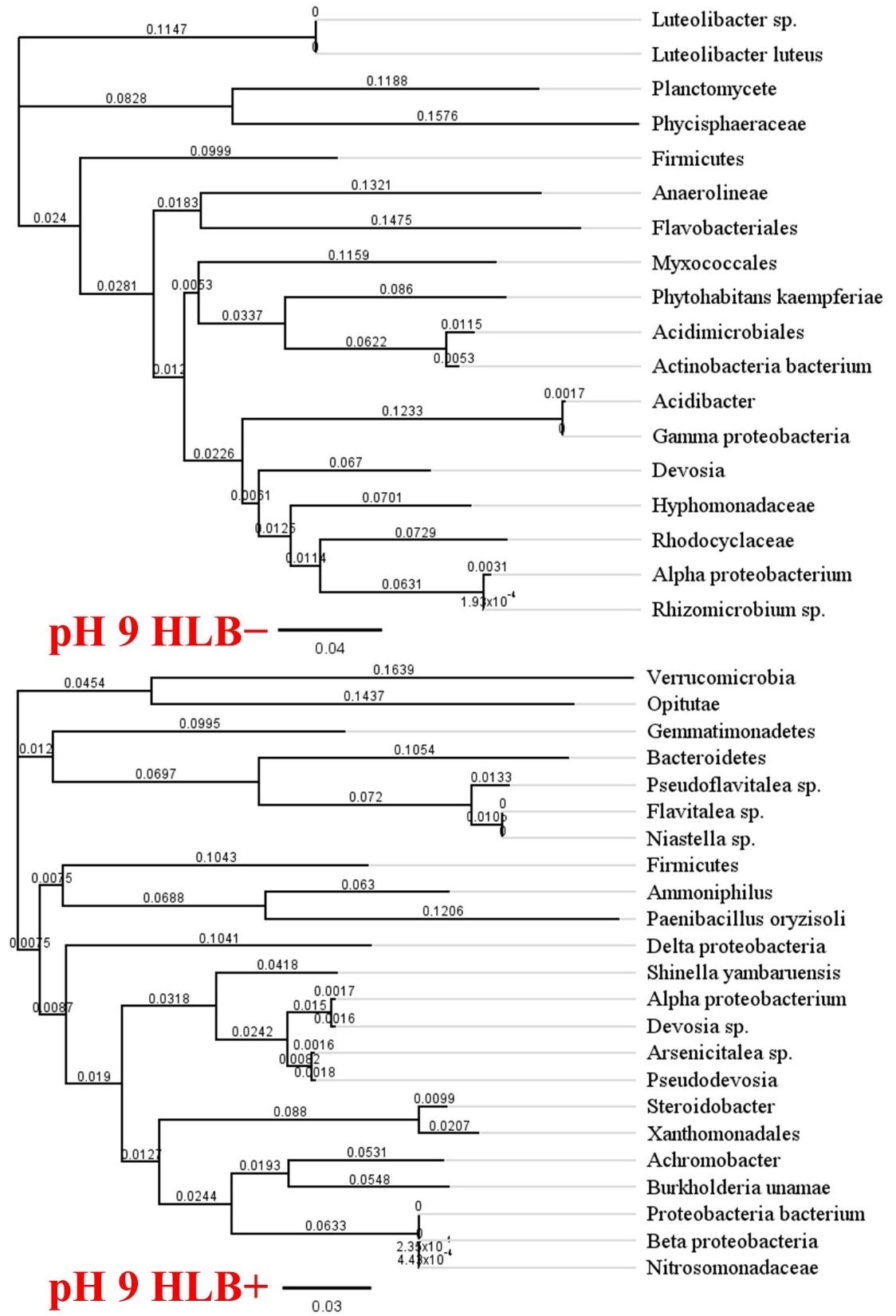


**Figure S6**. Bacteria phylogenetic analysis of soilless substrate under treatment pH 9 based on root microbe 16S rRNA sequencing of HLB− (top) and HLB+ (bottom) plants of ‘Ray Ruby’ grapefruit (*Citrus paradisi*) on Sour orange (*Citrus aurantium*) rootstock. The phylogenetic tree was built utilizing the neighbor-joining analysis method with bootstrap support at the nodes (Saitou & Nei, 1987).
